# The skin allergy risk assessment-integrated chemical environment (SARA-ICE) defined approach to derive points of departure for skin sensitization

**DOI:** 10.1016/j.crtox.2024.100205

**Published:** 2024-12-14

**Authors:** Emily N. Reinke, Joe Reynolds, Nicola Gilmour, Georgia Reynolds, Judy Strickland, Dori Germolec, David G. Allen, Gavin Maxwell, Nicole C. Kleinstreuer

**Affiliations:** a Inotiv, Inc., Morrisville, NC 27560, USA; b Unilever Safety and Environmental Assurance Centre, Colworth Science Park, Sharnbrook, Bedforshire MK44 1LQ, United Kingdom; c National Institute of Environmental Health Sciences, Division of Translational Toxicology, National Toxicology Program Interagency Center for the Evaluation of Alternative Toxicological Methods, P.O. Box 12233, Research Triangle Park, NC 27709, USA

**Keywords:** Skin sensitization, Non-animal methodology, Risk assessment, LLNA, Point of departure, Bayesian, Defined approach, Computational toxicology, Defined approach for skin sensitization

## Abstract

Mechanistically based non-animal methods for assessing skin sensitization hazard have been developed, but are not considered sufficient, individually, to conclusively define the skin sensitization potential or potency of a chemical. This resulted in the development of defined approaches (DAs), as documented in OECD TG 497, for combining information sources in a prescriptive manner to provide a determination of risk or potency. However, there are currently no DAs within OECD TG 497 that can derive a point of departure (POD) for risk assessment. The Skin Allergy Risk Assessment – Integrated Chemical Environment (SARA-ICE) DA for skin sensitization is a Bayesian statistical model that estimates a human-relevant metric of sensitizer potency, the ED_01_, an estimate of the human predictive patch test dermal dose at which there is 1% chance of inducing sensitization, which can be used in a risk assessment paradigm. The model accounts for variability of input data and explicitly quantifies uncertainty. SARA-ICE derives the ED_01_ from a variety of *in vitro* and *in vivo* test method data and is built upon historical human, murine, and *in vitro* test data for 434 chemicals. In addition to the ED_01_ POD SARA-ICE DA also provides a Globally Harmonized System of Classification and Labelling of Chemicals (GHS) classification probability for GHS subcategories 1A, 1B and not classified (NC). Here we describe the SARA-ICE model and its evaluation, including performance versus benchmark PODs. In addition, via a case study with isothiazolinones (ITs), we demonstrate the utility of SARA-ICE for integrating different data inputs and compare the ED_01_ for six ITs to existing historical data.

## Highlights

• Defined approach for skin sensitization for point-of-departure estimate.
• Bayesian statistical model with flexibility for decision making and data inputs.
• Isothiazolinone case study to compare performance with other defined approaches.
• SARA-ICE performed well correctly assigning GHS category except where only *in vivo* data were used as inputs.
• SARA-ICE median ED01 estimates were equivalent to reference values; *in vitro* data inputs were generally more conservative.

## Graphical abstract

**Figure:**
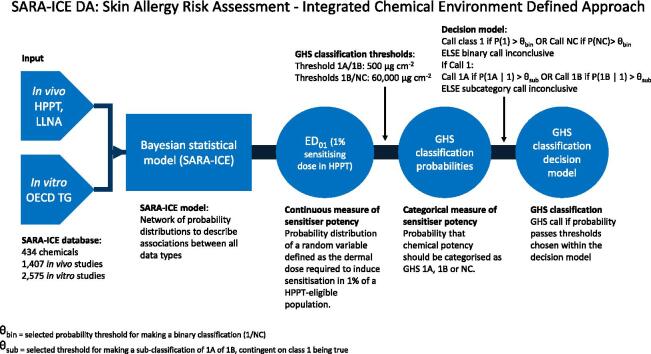

## Introduction

Regulatory assessment for the potential of chemicals to cause skin sensitization has historically relied on animal methods such as the murine local lymph node assay (LLNA), guinea pig maximization test (GPMT) or the Buehler test (OECD, 2022, OECD, 2010). Recent developments in the use of new approach methodologies (NAMs), developed in line with the human biology-based adverse outcome pathway (AOP) for skin sensitization and integrated into defined approaches (DAs), have allowed for the reduction or elimination of the traditional animal tests across multiple sectors (EPA, 2018, OECD, 2021, Kleinstreuer et al., 2018, OECD, 2014). DAs rely upon fixed data interpretation procedures which are applied to data from a pre-defined set of information sources (such as *in vitro, in chemico*, or *in silico* approaches) to derive a prediction without relying on expert judgment. Several DAs have been adopted by the Organisation for Economic Cooperation and Development (OECD), both for hazard assessment and potency sub-categorization according to the Globally Harmonized System of Classification and Labelling of Chemicals (GHS) (OECD, 2021). These existing DAs rely on adopted OECD test guideline methods as information sources, such as the direct peptide reactivity assay (DPRA), the KeratinoSens™, the human cell line activation test (h-CLAT), as well as two *in silico* hazard prediction models, DEREK Nexus™ and the OECD QSAR toolbox (OECD, 2022, OECD, 2022, OECD, 2022, Chilton et al., 2018, Yordanova et al., 2019). None of these DAs have been adopted for deriving a quantitative point of departure (POD) that provides accurate human-relevant potency information for skin sensitization risk assessment. Until recently, POD derivation relied on the animal methods listed above or human data from predictive patch tests (Strickland et al., 2023). Quantitative risk assessment for skin sensitization was initially developed to derive safe levels of fragrance materials for use in consumer products. In this context, it has been refined to incorporate aggregated exposures for fragrances and its use has also been extended to preservatives and pesticides (Gerberick et al., 2001, Griem et al., 2003, Api et al., 2020).

A comprehensive review of the international regulatory requirements for skin sensitization is provided in Daniel et al., 2018 (Daniel et al., 2018, Basketter et al., 2020). With the subsequent acceptance of the Defined Approaches for Skin Sensitization test guideline by OECD in 2021, the acceptance of combined approaches for assessment of hazard and potency under GHS came under the principles of mutual acceptance of data (MAD) (OECD, 2021, OECD, 1981). The principles of MAD dictate that OECD member countries or adherents to MAD must accept study data produced by other member countries under the purview of OECD test guidelines. Global regulatory acceptance of the DAs varies, with European Union regulatory agencies requiring the use of the DAs under most cases. Other countries, such as the U.S., accept them as part of a waiver or under interim policies (EPA, 2018, EC, 2016, EC, 2006). Other regions, such as China, are moving towards acceptance of the DAs (NIFDC, 2024). Where potency information is required, and particularly where animal methods are banned, such as with cosmetics assessments within the European Union, there is a need to evaluate data from newly developed methods within a framework where PODs can be identified. Accordingly, with recent developments in technologies, the use of non-animal methods in conjunction with computational models to produce PODs for skin sensitization risk assessment are being developed. Examples of such approaches include statistical regression models, machine learning and Bayesian models (Zang et al., 2017, Natsch and Gerberick, 2022, Jaworska et al., 2015, Tsujita-Inoue et al., 2015, Reynolds et al., 2019, Gilmour et al., 2022).

One specific example is the Skin Allergy Risk Assessment Integrated Chemical Environment (SARA-ICE) defined approach. SARA-ICE is used for deriving a probabilistic human-relevant POD which can be applied to GHS skin sensitization potency categories and hazard assessment. The SARA-ICE defined approach is a Bayesian model based on the prototype SARA model developed by J Reynolds *et al* in 2019 (Reynolds et al., 2019). The original model estimated chemical-specific maximum exposure levels that are non-sensitizing for individuals in a human repeat insult patch test (HRIPT)-eligible population. Model parameters were estimated from a database of 30 skin sensitizers with data from the HRIPT, LLNA, DPRA, KeratinoSens, h-CLAT, and U-SENS™. The model and underlying database were subsequently revised and expanded (from 30 to 81 chemicals) and the POD was changed to an estimate of a 1 % sensitizing dose for individuals in a HRIPT-eligible population (ED_01_). A second output was added to derive a risk metric, defined as the probability that an exposure to an ingredient was low risk, by incorporating high- and low-risk consumer goods risk benchmarks into the model. To further broaden the applicability of the SARA model to wider range of chemical types and to increase its application across industry and regulatory needs, a collaboration between Unilever and the National Toxicology Program Interagency Center for the Evaluation of Alternative Toxicological Methods (NICEATM) was initiated to leverage the ICE database and create the SARA-ICE DA, encompassing data for 434 chemicals. The model has been made available as a locally executable program and will be housed within the ICE platform (https://ice.ntp.niehs.nih.gov).

The SARA-ICE DA is therefore shared in full, including model specifications. To demonstrate the model is robust, we have described a series of model checks alongside an evaluation of the model’s predictive performance versus the established OECD reference dataset of GHS classifications for 196 compounds (OECD, 2021). We further assessed the robustness of the performance statistics by using a cross-validation approach.

Assessing the applicability of new models for deriving PODs often rely on case studies for different chemical groups, as has already been conducted for the SARA model (OECD. Annex I: 2016, OECD, 2022). Isothiazolinones (ITs, Table 1) are a family of preservatives found in a wide variety of consumer use products. These products, which include household cleaning products, latex paints, pressure-treated wood, metal working fluids, textiles, and plastics, are an ideal chemical group to use as a case study to demonstrate applicability of SARA-ICE (Basketter et al., 1999, Silva et al., 2020). Regional regulatory requirements for skin sensitization assessment of ITs vary throughout the world and are dependent upon their use-case. In this case study, using previously published data generated in OECD-adopted *in vitro* and *in chemico* test methods, we demonstrate application of SARA-ICE to derive human-relevant PODs for risk assessment and an associated potency categorization (Gilmour et al., 2022). We compared these SARA-ICE DA outputs to existing historical animal data, alternative DAs, and, where available, human relevant PODs derived from a weight-of-evidence approach.

**Table 1:** Summary of key differences between published SARA models and SARA-ICE Model.

	SARA prototype(Reynolds et al. 2019)	SARA model (Reynolds et al. 2022)	SARA-ICE
**Database**	**30 chemicals, 892 studies**	**81 chemicals, 1200 studies**	**434 Chemicals, >4000 studies**
**Assay Inputs**	**HRIPT, LLNA, DPRA, KeratinoSens, hCLAT, U-SENS**	**SARA Prototype + consumer goods risk benchmarks**	**SARA Prototype + kDPRA (log kmax), HMT, cytotoxicity concentrations from KeratinoSens, hCLAT, U-SENS**
**Probability of Sensitiser**	Assumes sensitizer*	Assumes sensitizer*	**Probability of GHS NC / 1 (binary)**
**Production Model**	**N/A**	**N/A**	**Faster production model (to be hosted on ICE)**
**Probability of GHS Cat.**	**N/A**	**N/A**	Probability of GHS Cat., *binary or 1A, 1B, NC
**Risk Model**	**N/A**	**Probability exposure to ingredients in consumer products is low risk**	**N/A**

*Assumes sensitizer – it is explicitly assumed that a chemical is a sensitizer when using the model.

GHS NC = Not classified, 1 = Sensitizer, 1A = high frequency of sensitizing occurrence/high potency, 1B = moderate frequency of sensitizing occurrence/low to moderate potency.

## Methods

The SARA-ICE DA is a Bayesian statistical model based upon the SARA Model, which estimates a POD, the ED_01,_ using any available combination of model inputs (Reynolds et al., 2019). The ED_01_ (units µg cm^−2^) from the SARA-ICE DA is an estimate of the human predictive patch test (HPPT) dermal dose at which there is 1 % chance of inducing sensitization. SARA-ICE is predicated on a dataset of unprecedented size and quality that was established by combining data from Reynolds et al., 2022 with available LLNA and *in vitro* reference data from ICE, and human reference data from a curated dataset of human maximization test (HMT) and HRIPT data (collectively referred to as HPPT) (Strickland et al., 2023, Bell et al., 2017). The HPPT data were curated in support of validating the DAs contained in OECD TG 497 (OECD, 2021). Reference models for inclusion in the SARA-ICE database were based on the most comprehensive collated data within the starting data sets, additional models for inclusion may be considered at a future time. A number of enhancements beyond the SARA model have been introduced, which are summarized in Table 1, along with the types of data that can be used to predict the ED_01_. These include a database of 434 chemicals substantially extended beyond cosmetics ingredients, increased assay input options including *in chemico* (DPRA, kinetic (k)DPRA)*, in vitro* (KeratinoSens, h-CLAT, U-SENS), and *in vivo* (HRIPT, HMT, LLNA) test methods, and probability estimates of GHS classification. These enhancements provide greater application of the DA across a larger chemical set, greater data use flexibility, and direct application to a wider range of regulatory requirements. The model is conditioned on this expanded database, which results in a posterior distribution of model parameters. The posterior distribution is used to define the parameters of a production version of SARA-ICE, allowing users to input chemical-specific data to rapidly estimate the ED_01_ for a chemical of interest. The risk model contained in the Reynolds et al. 2019 SARA publication (Reynolds et al., 2019) has been removed from the SARA-ICE model to provide an objective, quantitative POD that can be used across risk assessment frameworks and jurisdictions.

An estimate of the ED_01_ uses a probability distribution as a centered 90 % credible interval (interval between the 5th and 95th percentiles of the distribution). Location-like summary statistics, such as the mean or median of the distribution represent the “best estimate”, whilst the variance of the distribution represents the uncertainty in the estimate. A conservative estimate of the ED_01_, which considers X% of the uncertainty in the estimate, can be obtained by using a suitably low percentile of the distribution (e.g. 5th percentile). The degree of conservatism is measured by the fold-difference between the median (50th percentile) and the 5th percentile. We call this measure of uncertainty inherent to an ED_01_ estimate the *SARA-ICE prediction uncertainty ratio* (SPUR). Several factors influence the SPUR, including the number of inputs, type of inputs, and concordance between inputs. The uncertainty in each ED_01_ estimate is unique and calculated dynamically based on the available inputs.

The second output of the SARA-ICE DA is a set of GHS classification probabilities for GHS subcategories 1A, 1B and not classified (i.e., nonsensitizer) (NC). These are obtained by setting the ED_01_ probability distribution thresholds at 500 µg cm^−2^ and 60,000 µg cm^−2^, which are the GHS 1A and 1B dose thresholds for the HPPT-derived dose per unit area (DSA), and by calculating the probability mass on either side and between these thresholds (United Nations, 2023).

Assessment of GHS classification performance requires us to choose a confidence threshold to translate classification probabilities into actual classifications (see SI 1 for details of the decision model). Let $\theta_{\text{bin}}$ denote the binary classification threshold and $\theta_{\text{sub}}$ denote the subcategory threshold. To choose appropriate values for these, we first note that in the absence of chemical-specific information to inform the ED_01_ estimate, GHS classification probabilities are simply equal to the prior mass assigned to each GHS classification, which is a uniform distribution over the GHS subcategories 1A, 1B and NC. Thus, the prior distribution for binary class 1 has a probability mass of 2/3 ≈ 0.67 and 0.50 for the subcategories 1A | 1 and 1B | 1 (conditional probabilities given that a binary call of 1 is made). It seems prudent to stipulate that classifications should not be possible in the absence of chemical-specific information, therefore appropriate binary and subcategory classification thresholds should be $\theta_{\text{bin}}=0.67+{∊}_{\text{bin}}$ and $\theta_{\text{sub}}=0.50+{∊}_{\text{sub}}$ for some choice of parameters ${∊}_{\text{bin}},{∊}_{\text{sub}}>0.$ There is a trade-off between classification performance and the rate of inconclusive predictions. Choosing ${∊}_{\text{bin}}$ and ${∊}_{\text{sub}}$ such that $\theta_{\text{bin}}$ and $\theta_{\text{sub}}$ are close to one will result in accurate classifications where they can be made, however at considerable cost in terms of the number of inconclusive classifications. Given this consideration we propose the criteria that probability thresholds for classification are a fixed number of units on the log-odds scale above their prior probabilities. For example, if we stipulate that thresholds are 0.5 logits above prior probabilities, then^3^ $\theta_{\text{bin}}=\text{logi}{\text{t}}^{\text{-1}}\left(\text{logit}\left(0.67\right)+0.5\right)\approx 0.77$ and $\theta_{\text{sub}}=\text{logi}{\text{t}}^{\text{-1}}\left(\text{logit}\left(0.50\right)+0.5\right)\approx 0.62$. These thresholds are used to assess SARA-ICE classification performance against benchmark GHS classifications. As described in the SARA-ICE Technical Specification supplementary information, the SARA-ICE model estimates GHS classification probabilities. The probability of NC is compared against a binary threshold $\theta_{\text{bin}}$. If the probability exceeds this threshold, a call of NC is made. If a call of NC cannot be made, then the probability of class 1 (one minus the probability of NC) is compared against the same binary threshold $\theta_{\text{bin}}$. If the probability of class 1 exceeds the threshold, a call of class 1 is made. If neither binary call is made, as would be the case if either the probability of NC or class 1 are between $1-\theta_{\text{bin}}$ and $\theta_{\text{bin}},$ then the binary call is inconclusive. In short, for a hazard classification, a chemical is class 1P(1) > *θ_bin_* and is NC if P(NC) > *θ_bin_*_._, otherwise, the call is inconclusive. If class 1 is called, conditional subcategory probabilities of 1A and 1B given class 1 are compared against the subcategory threshold $\theta_{\text{sub}}$ such that a chemical is 1A if P(1A | 1) > *θ_sub_* or 1B if P(1B | 1) > *θ_sub_*. If neither probability exceeds the threshold, the subcategory call is inconclusive, but the chemical is still class 1.

Complete specification of the model includes mathematical specification of the probability distributions assumed. These details are provided, in full, in the supplementary file 1 “SARA-ICE_Technical_Specification.docx”.

Model Evaluation

The SARA-ICE model was evaluated, firstly, using posterior predictive checks as is recommended by Gelman *et al.* for any Bayesian statistical model (Gelman et al., 1995). Briefly, checks performed assessed whether model parameters were identifiable from the database and whether inferred associations between input types matched prior expectations. Checks were also run to assess whether the SARA-ICE production model gave the same output as the full model.

Secondly, predictive performance of the GHS classification component of the model was benchmarked against a reference set of GHS classifications established by the OECD (OECD, 2021). This replicated the steps used to evaluate other defined approaches for skin sensitization (OECD, 2021).

Application of SARA-ICE to the ITs.

We selected six ITs from those evaluated in a previous draft risk assessment by the EPA and NICEATM (Table 3) (Strickland et al., 2022): 2-n-Octyl-4-isothiazolin-3-one (OIT), 2-Methyl-4-isothiazolin-3-one (MIT), Mixture of 5-chloro-2-methyl-4-isothiazolin-3-one and 2-methyl-4-isothiazolin-3-one (CMIT/MIT), 1,2-Benzisothiazolin-3-one (BIT), 4,5-Dichloro-2-octyl-3(2H)-isothiazolone (DCOIT) and 1,2-Benzisothiazolin-3-one, 2-butyl (BBIT).

**Table 2:** Summary of study/assay counts in the SARA and SARA-ICE databases.

	SARA prototype	SARA	SARA-ICE
Unique CASRN	**30**	**81**	**434**
HRIPT	**103**	**143**	**365**
HMT	**0**	**0**	**506**
LLNA	**134**	**266**	**536**
DPRA	**240**	**281**	**650**
kDPRA	**0**	**0**	**361**
KeratinoSens	**193**	**210**	**972**
h-CLAT	**95**	**148**	**431**
U-SENS	**127**	**152**	**164**
Benchmark Exposure	**0**	**152**	**0**

**Table 3:** Isothiazolinones considered in this study.

**Chemical Name** **(Abbreviation; CASRN)**	**MW**	**Chemical Structure**	**Input data**					**Reference Data**			
			**DPRA**^1^**(% depletion)**	**KeratinoSens^2^**	**h-CLAT^3^**	**LLNA^4^**	**HPPT^5^**	**NESIL** **(µg cm-2)**	**GHS^6^**	**EPA POD^7^ (µg cm-2)**	**LLNA EC3^8^** **(µg cm-2)**
MIT (2-Methyl-4-isothiazolin-3-one; 2682–20-4	115.16		Cys: 100 % Lys: 0 %	EC1.5: 9.54 µM IC50: 108 µM	CD54 EC200: 11.6 µg/ml CD86 EC150: 11.8 µg/ml CV75: 24.6 µg/ml	EC3: 0.4 % to > 4.5 % (4 studies)	DSA: 10 µg cm-2 to 30 µg cm-2 Ntested: 75 to 210 Nsensitised: 0 to 1 (6 studies)	15^9^	1A	210	288
CMIT/MIT (Mixture of 5-chloro-2-methyl-4-isothiazolin-3-one and 2-methyl-4-isothiazolin-3-one; 55965–84-9)	141.36^12^		Cys: 100 % Lys:: 10.6 %	EC1.5: 3.41 µM IC50: 19.9 µM	CD54 EC200: 2.63 µg/ml CD86 EC150: 2.81 µg/ml CV75: 3.04 µg/ml	EC3: 0.0049 % to 0.048 % (9 studies)	DSA: 0.83 µg cm-2 to 79 µg cm-2 Ntested: 45 to 602 Nsensitised: 0 to 7 (13 studies)	0.83^10^	1A	120	4.5
BIT (1,2-Benzisothiazolin-3-one; 2634–33-5)	151.18		Cys: 100 % Lys: 0 %	EC1.5: 3.14 µM IC50: 57.8 µM	CD54 EC200: 7.63 µg/ml CD86 EC150: 7.84 µg/ml CV75: 13.1 µg/ml	EC3: 1.5 % to 32.4 % (7 studies)	DSA: 45 µg cm-2 to 91 µg cm-2 Ntested: 54 to 58 Nsensitised: 0 to 5 (2 studies)	45^11^	1	85	2642
OIT (2-n-Octyl-4-isothiazolin-3-one; 26530–20-1)	213.34		Cys: 100 % Lys: 1.3 %	EC1.5: 2.19 µM IC50: 12.7 µM	CD54 EC200: 0.95 µg/ml CD86 EC150: 7.26 µg/ml CV75: 8.8 µg/ml	EC3: 0.2 % to 0.66 % (4 studies)		2.7^12^	1A	3.75	90.25
DCOIT (4,5-Dichloro-2-octyl-3(2H)-isothiazolone; 64359–81-5)	282.23		Cys: 100 % Lys: 11.6 %	EC1.5: 1.32 µM IC50: 4.65 µM	CD54 EC200: 0.92 µg/ml CD86 EC150: >1.081 µg/ml CV75: 0.9 µg/ml	EC3: 0.0041 % to 0.011 % (2 studies)		6.3^12^	1A	5.8	2
BBIT (1,2-Benzisothiazolin-3-one, 2-butyl; 4299–07-4)	207.29		Cys: 100 % Lys: 0 %	EC_1.5_: 3.84 µM IC_50_: 53.0 µM	CD54 EC_200_: 3.01 µg/ml CD86 EC_150_: 3.15 µg/ml CV_75_: 3.3 µg/ml			N/A	1	15	N/A

1 NICEATM IT report, Appendix A, Table 2; ^2^ NICEATM IT report, Appendix A, Table 5; ^3^ NICEATM IT report, Appendix A, Tables 7 & 8; ^4^ NICEATM IT report, Appendix C; ^5^Strickland et al., 2023, Herzler et al., 2024; ^6^ECHA; ^7^ EPA DOCKET (https://www.regulations.gov/document/EPA-HQ-OPP-2017–0720-0011); ^8^Converted to µg cm-2 by multiplying average of values from LLNA column by 250 per Griem et al., 2003; ^9^EC, 2015; ^10^Burnett et al., 2021; ^11^Novick et al., 2013; ^11^Ladics et al., 2020; ^13^Average molecular weight assuming a CMIT:MIT ratio of 0.761:0.239.

To evaluate the performance of SARA-ICE DA as applied to the ITs, we used different combinations of input data from both *in vitro* and *in vivo* sources. In a previous study, data on IT skin sensitization hazard came from the DPRA, KeratinoSens, and the h-CLAT (Strickland et al., 2022). These data, along with compiled *in vivo* data from the LLNA and HPPT data, were used as variable inputs for the model. All input ranges are listed in Table 3.

For each of the six IT chemicals considered here, the SARA-ICE DA was used to estimate the ED_01_ and GHS classification using the following combinations of data:

1. NAM inputs only (1 x DPRA + 1 x KeratinoSens + 1 x h-CLAT).
2. LLNA inputs only (except BBIT).
3. HPPT inputs only (CMIT/MIT, MIT, and BIT only).
4. NAM and LLNA inputs (except BBIT).
5. NAM and HPPT inputs (CMIT/MIT, MIT, and BIT only).
6. LLNA and HPPT inputs (CMIT/MIT, MIT, and BIT only).
7. NAM, LLNA and HPPT inputs (CMIT/MIT, MIT, and BIT only).

Three of the six isothiazolinone chemicals were present in the SARA-ICE database. For the purposes of this case study, these three chemicals were removed when estimating model parameters, reducing the training dataset size from 434 to 431 unique CASRN.

Reference Data for Comparison to SARA-ICE results

To evaluate the performance of SARA-ICE, reference values such as no-expected-sensitization-induction levels (NESILs) and GHS categorizations were obtained from primary literature or regulatory evaluations (NESILs) or from the European Chemicals Agency (ECHA) Chemicals Database (GHS), see Table 3. We also included EPA PODs that were derived from an artificial neural network model (ANN) for skin sensitization with *in vitro* inputs, which predicts an LLNA EC3 value (Strickland et al., 2022, ECHA. European Chemicals Agency, 2023). The EC3 is the percent concentration at which a stimulation index of three, the positive threshold response, is induced in the mouse LLNA. Reference LLNA values are also provided, which have been converted to a dose-per-skin area value. This conversion from an EC3 to a dose-per-skin area value is accomplished by multiplying the EC3 by 250 to account for the approximate surface area of the mouse ear (1 cm^2^) and to convert from percent to volume (10 to convert to µg/µL and 25 µl to account for the volume applied) (Griem et al., 2003). Table 3 provides a summary of the reference values for evaluation.

## Results and discussion

Model evaluation

The reliability of inferences from statistical models is highly dependent on the degree of concordance between the model and data and therefore basic model checks were performed. These results are documented in full in the SARA-ICE Technical Specification supplementary information.

**Fig. 1:**
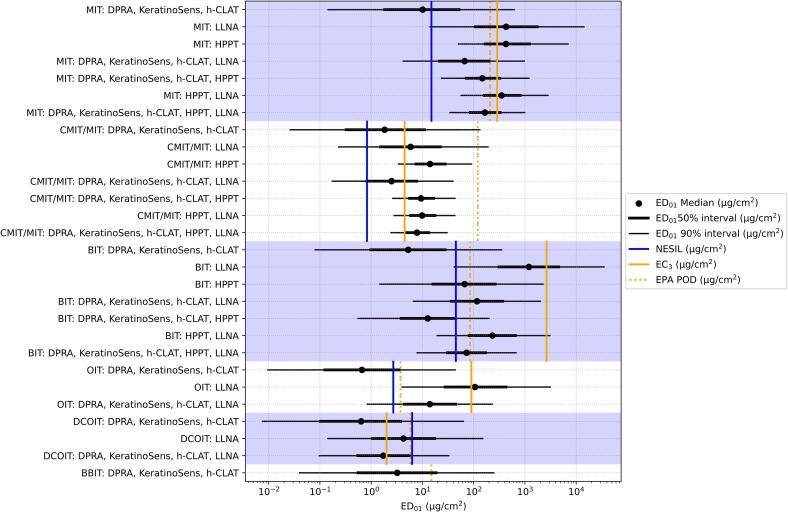
ED_01_ estimates represented as centered 90% credible intervals (thin line), 50% credible intervals (thick line) and median (bullet). From Table 3, blue lines indicate the reference NESIL, dashed orange lines are plotted at the EPA POD and solid orange lines are plotted at the reference LLNA EC3.

The first model check showed that key model parameters were identifiable from the SARA-ICE database, and furthermore, were not unduly influenced by prior distribution choices. Fig. 2 in Supplementary Information 1 (SI 1) shows that posterior estimates of key model parameters are not concentrating in the tails of the priors, indicating each parameter is inferable from the database. The next set of model checks looked at the correlations between the ED_01_ and other input types; from SI 1 Fig. 1, observe that the model infers reasonably strong correlations between the ED_01_ and latent model parameters specific to non-HPPT input types (Pearson correlation coefficients range from 0.70 to 0.92 across the various input types). For the KeratinoSens^TM^, h-CLAT and U-SENS^TM^ assays, correlations with assay-specific outputs were higher than correlations with cytotoxicity measures. We also computed correlations between the non-HPPT inputs. Fig. 4 in SI 1 shows that the highest correlations occur between similar inputs: for example, the highest correlations were generally found between cytotoxicity measures from the three cell-based assays. In addition, the DPRA input was highly correlated with kinetic DPRA reactivity measure. These observations matched what we expected *a priori* and therefore serve to boost confidence in model performance. Fig. 5 in SI 1 shows that latent variables correlated with the ED_01_ are themselves highly correlated with assay-specific inputs. Fig. 6 in SI 1 illustrates the overall association between assay-specific inputs and the ED_01_.

Finally, ED_01_ estimates obtained from the full SARA-ICE model were compared against those obtained from the SARA-ICE production model to check that both models are returning the same inferences. Fig. 7 in SI 1 shows that correlations between percentiles were very close to one, indicating that both models give the same output for all practical purposes.

GHS Classification Performance of SARA-ICE.

Within the OECD reference dataset, 66/196 chemicals are assigned a binary GHS classification of 1 or NC based on human data, and 52/66 of these are assigned a GHS subcategory of 1A or 1B. There are 168/196 chemicals with an assigned binary GHS classification based on LLNA data with 123/168 assigned a subcategory classification of either 1A or 1B. Predictive performance of the SARA-ICE model was assessed against this reference dataset by restricting inputs to the NAM inputs in Annex 2 of the supporting document for TG 497 and an additional kDPRA input sourced from Natsch & Gerberick, 2022 (Natsch and Gerberick, 2022, OECD, 2021).

Binary classification performance after setting $\theta_{\text{bin}}=0.77$ (see Methods) is reported in Table 4. In order to apply these thresholds, a binary call of 1 or NC is made if the probability of either 1 or NC exceeds the 0.77 probability threshold $\left(\theta_{\text{bin}}\right)$. If a chemical exceeds the 0.77 threshold for category 1, the chemical is considered sensitizer; a subcategorization assessment can then be made, dependent on the probability of a GHS 1A or 1B classification exceeding the 0.62 probability threshold ${(\theta }_{\text{sub}})$. The classification is *inconclusive* if either the binary classification probability for Class 1 or NC fails to exceed the threshold. Classification performance statistics are computed for the subset of chemicals within which a conclusive call is made. Balanced accuracy for binary classifications exceeds 90 % for both human and LLNA benchmarks. Subcategory classification performance is reported in Table 5. Performance for Class NC is similar to the binary case (the number of chemicals classified as NC does not change at the subcategory level). For chemicals with a binary classification of 1, the SARA-ICE model attempts to make a subcategory call of either 1A or 1B using the subcategory threshold $\theta_{\text{sub}}=0.62$. Average balanced accuracy across the subcategories is around 82 %/83 % for human/LLNA benchmarks. Inconclusive rates for each benchmark class are also reported in Table 4 and Table 5. Inconclusive rates tend to be lower for Class 1 on the binary decision and lower for Class 1A on the subcategory decision.

**Table 4:** Binary classification performance of the SARA-ICE model against reference GHS classifications.

**Human, Θ_bin_ = 0.77**	**SARA-ICE 1**	**SARA-ICE NC**	**Inconclusive**	**Total**
Reference (OECD, 2022)	37	5	13	55
**Reference NC**	0	5	6	11
**Total**	37	10	19	66
Sensitivity: 88 % Specificity: 100 % **Balanced accuracy: 94 %** Inconclusive rate on reference class 1: 24 % Inconclusive rate on reference class NC: 55 %				
**LLNA, Θ_bin_ = 0.77**	**SARA-ICE 1**	**SARA-ICE NC**	**Inconclusive**	**Total**
Reference (OECD, 2022)	89	9	37	135
**Reference NC**	2	19	12	33
**Total**	91	28	49	168
Sensitivity: 91 % Specificity: 90 % **Balanced accuracy: 91 %** Inconclusive rate on reference class 1: 27 % Inconclusive rate on reference class NC: 36 %

**Table 5:** Subcategory classification performance of the SARA-ICE model against reference GHS classifications.

**Human, Θ_bin_ = 0.77, Θ_sub_ = 0.62**	**SARA**-**ICE 1A**	**SARA-ICE 1B**	**SARA**-**ICE NC**	**Inconclusive**	**Total**
Reference (OECD, 2022)**A**	14	2	0	5	21
Reference (OECD, 2022)**B**	3	7	5	16	31
**Reference NC**	0	0	5	6	11
**Total**	17	9	10	27	63
Sensitivity 1A: 88 %, Specificity 1A: 85 %, Balanced accuracy 1A: 86 % Sensitivity 1B: 47 %, Specificity 1B: 90 %, Balanced accuracy 1B: 69 % Sensitivity NC: 100 %, Specificity NC: 84 %, Balanced accuracy NC: 92 % **Average balanced accuracy: 82 %** Inconclusive rate on reference class 1A: 24 % Inconclusive rate on reference class 1B: 52 % Inconclusive rate on reference class NC: 55 %					
**LLNA, Θ_bin_ = 0.77, Θ_sub_ = 0.62**	**SARA-ICE 1A**	**SARA-ICE 1B**	**SARA-ICE NC**	**Inconclusive**	**Total**
Reference (OECD, 2022)**A**	27	3	0	8	38
Reference (OECD, 2022)**B**	12	22	8	43	85
**Reference NC**	0	1	19	13	33
**Total**	39	26	27	64	156
Sensitivity 1A: 90 %, Specificity 1A: 81 %, Balanced accuracy 1A: 85 % Sensitivity 1B: 52 %, Specificity 1B: 92 %, Balanced accuracy 1B: 72 % Sensitivity NC: 95 %, Specificity NC: 89 %, Balanced accuracy NC: 92 % **Average balanced accuracy: 83 %** Inconclusive rate on reference class 1A: 21 % Inconclusive rate on reference class 1B: 51 % Inconclusive rate on reference class NC: 39 %					

The confidence thresholds used here offer a reasonable balance between classification accuracy and the inconclusive rate. In SI 2 we provide a more comprehensive analysis in which we 1. explore classification performance of the SARA-ICE model when making predictions using all available data in the SARA-ICE database, 2. examine stability of classifications using NAM inputs to perturbations to the training set of the model using a cross-validation procedure and 3. report classification performance for different choices of decision thresholds.

Output of SARA-ICE for the ITs

For each IT, the ED_01_ was generated using different combinations of input data derived from NAMs or historical *in vivo* data (LLNA or HPPT data) (Table 3 and Supplemental Table 1, Table 2). When using *in chemico/in vitro* inputs only, the median ED_01_ estimate ranges from 0.64 µg cm^−2^ (DCOIT) to 10 µg cm^−2^ (MIT). The 5th percentile of the distribution ranges from 0.0076 µg cm^−2^ (DCOIT) to 0.14 µg cm^−2^ (MIT). The degree of uncertainty in an estimate is summarized using the *SARA-ICE prediction uncertainty ratio* (SPUR, see Methods for complete specification), where lower values indicate lower levels of uncertainty. For this combination of inputs, the SPUR ranges from 65 to 81 across the six ITs. Estimates of the ED_01_ obtained using the SARA-ICE model are provided in Table 4. A visual summary of these estimates and their associated uncertainty is provided in Fig. 1. Table 6 provides all estimates of the ED_01_ dependent on input data; median ED_01_ values are highlighted in grey.

**Table 6:** ED01 estimates obtained using SARA-ICE.

**Chemical**	**Input Data**	**ED_01_ percentiles (μg cm^−2^)**			SPUR* (50th / 5th)
		**5th**	**50th**	**95th**	
MIT	DPRA, KeratinoSens, h-CLAT	0.14	10	6.1e + 02	70
MIT	LLNA	14	4.3e + 02	1.4e + 04	31
MIT	HPPT	51	4.3e + 02	7e + 03	8.4
MIT	DPRA, KeratinoSens, h-CLAT, LLNA	4.2	67	9.7e + 02	16
MIT	DPRA, KeratinoSens, h-CLAT, HPPT	24	1.5e + 02	1.2e + 03	6.3
MIT	HPPT, LLNA	57	3.5e + 02	2.8e + 03	6.1
MIT	DPRA, KeratinoSens, h-CLAT, HPPT, LLNA	34	1.6e + 02	9.9e + 02	4.8
CMIT/MIT	DPRA, KeratinoSens, h-CLAT	0.026	1.8	1.3e + 02	70
CMIT/MIT	LLNA	0.23	5.9	1.9e + 02	25
CMIT/MIT	HPPT	3.4	14	90	4.1
CMIT/MIT	DPRA, KeratinoSens, h-CLAT, LLNA	0.18	2.5	39	14
CMIT/MIT	DPRA, KeratinoSens, h-CLAT, HPPT	2.6	9.4	43	3.6
CMIT/MIT	HPPT, LLNA	2.8	9.9	43	3.5
CMIT/MIT	DPRA, KeratinoSens, h-CLAT, HPPT, LLNA	2.4	7.9	30	3.2
BIT	DPRA, KeratinoSens, h-CLAT	0.081	5.3	3.5e + 02	65
BIT	LLNA	42	1.2e + 03	3.5e + 04	29
BIT	HPPT	1.5	67	2.3e + 03	45
BIT	DPRA, KeratinoSens, h-CLAT, LLNA	6.7	1.2e + 02	2e + 03	17
BIT	DPRA, KeratinoSens, h-CLAT, HPPT	0.55	13	2e + 02	23
BIT	HPPT, LLNA	19	2.3e + 02	3.1e + 03	12
BIT	DPRA, KeratinoSens, h-CLAT, HPPT, LLNA	7.9	73	6.7e + 02	9.2
OIT	DPRA, KeratinoSens, h-CLAT	0.0096	0.66	43	68
OIT	LLNA	4	1.1e + 02	3.1e + 03	26
OIT	DPRA, KeratinoSens, h-CLAT, LLNA	0.84	14	2.3e + 02	17
DCOIT	DPRA, KeratinoSens, h-CLAT	0.0076	0.64	63	84
DCOIT	LLNA	0.14	4.3	1.5e + 02	30
DCOIT	DPRA, KeratinoSens, h-CLAT, LLNA	0.097	1.7	33	18
BBIT	DPRA, KeratinoSens, h-CLAT	0.04	3.2	2.5e + 02	81

* SPUR = SARA-ICE prediction uncertainty ratioGrey highlights the median values predicted by SARA-ICE.

For ED_01_ estimates obtained using LLNA data only, the median ED_01_ estimate ranges from 4.3 µg cm^−2^ (DCOIT) to 1,200 µg cm^−2^ (BIT). The 5th percentile of the estimate ranges from 0.14 µg cm^−2^ (DCOIT) to 42 µg cm^−2^ (BIT). The SPUR values when estimating the ED_01_ using multiple LLNA inputs are smaller than those with NAM inputs, ranging from 25 to 31.

For CMIT/MIT, MIT and BIT, HPPT inputs are available. Median ED_01_ estimates range from 14 µg cm^−2^ (CMIT/MIT) to 430 µg cm^−2^ (MIT). The number of HPPT studies per chemical is variable. CMIT/MIT has 13 HPPT studies which results in the lowest SPUR of 4.1, whereas MIT has six HPPT studies and a SPUR of 8.4. BIT has only two HPPT studies available; the SPUR uncertainty metric in this case is 45.

When estimated using combinations of data types (NAMs vs LLNA vs HPPT), ED_01_ values are more precise (have lower variance) than when estimated using isolated inputs. Furthermore, median ED_01_ estimates tend to be centered between estimates of isolated inputs. For example, the *in chemico/in vitro* median ED_01_ estimate for MIT is 10 µg cm^−2^ whilst the median LLNA estimate is 430 µg cm^−2^. After combining these inputs, the median estimate is 67 µg cm^−2^. The SPUR is 16 when inputs are combined and has values of 70 (*in chemical/in vitro*) and 31 (LLNA) for isolated inputs.

When estimating the ED_01_ using multiple input types, the SARA-ICE model combines them in a probabilistic fashion, where it is usually the case that additional inputs serve to reduce the uncertainty in the ED_01_ estimate. Consider the HPPT-only estimate of the ED_01_ for MIT, which has a median of 430 µg cm^−2^ and a SPUR uncertainty metric of 8.4. When the HPPT-estimates are combined with the LLNA data for MIT, the median ED_01_ estimate is 350 µg cm^−2^ and the SPUR is reduced to 6.1. The additional data in this case reduces the “best estimate” of the ED_01_, but also reduces the uncertainty associated with the input. The addition of *in chemico/in vitro* inputs in this case further reduces the best estimate to 160 µg cm^−2^ and the SPUR to 4.8. This trend was consistent across all of the ITs explored here, indicating that while partial information sources can provide adequate estimates, greater amounts of input data typically reduce uncertainty, resulting in an increased level of precision in the estimate of the ED_01_.

Comparison of SARA-ICE results to reference values.

The ED_01_ POD is intended for use in a quantitative risk assessment of skin sensitization for a given exposure scenario. This is dependent on the regulatory need and context of use and could potentially be used within a next-generation risk assessment (NGRA) framework (Gilmour et al., 2022, SCCS, 2023, Gilmour et al., 2020). Various POD types are already in use, such as human-relevant NESILs or the murine LLNA EC3 (Api et al., 2020). NESILs are derived from a weight-of-evidence approach, assessing all possible data, and require expert judgement to develop a dose per skin area value. Because these NESILs require expert judgement, they can be time-consuming to derive, and derivation may not be consistent across experts. This contrasts with the way a DA such as SARA-ICE, with a fixed data interpretation procedure, would derive a POD. However, NESILs and other PODs such as measured or predicted EC3s provide a useful comparison given their historical use within risk assessments. Application of the SARA-ICE derived PODs within a full risk assessment application will be explored within a subsequent publication.

The median ED_01_ estimates for different combinations of input data were compared to existing reference data, including NESILs, EPA PODs which were derived from the predicted EC3 outputs of an ANN model DA, and average LLNA EC3 reference values (Fig. 1) (Burnett et al., 2021, Novick et al., 2013, Strickland et al., 2022, European Commission EC, 2015, EPA, 2020, Ladics, 2020). BBIT does not have a reference NESIL or LLNA output, leaving five ITs for comparison. When using *in chemico/in vitro* data only, the median ED_01_ estimate is a conservative estimate of the NESIL (vertical red lines, Fig. 1) for four of the five ITs. The 5th percentile of the ED_01_ estimate is a conservative estimate of the NESIL for all five ITs. Alternatively, when estimated using all available *in vivo* data, the median ED_01_ estimate is greater than the NESIL for all ITs except DCOIT. The 5th percentile of the estimate is lower than the NESIL for DCOIT and BIT. Finally, when the ED_01_ is estimated using all available data (*in vivo* and in *in chemico/in vitro*), the median ED_01_ estimate is again greater than the NESIL for all ITs except DCOIT. However, with this data combination, the 5th percentile is lower for DCOIT, OIT and BIT.

For the purposes of deriving safe-exposure levels, SARA-ICE would provide a more protective POD for risk assessment than the NESIL when using ED_01_ estimates based on only NAMs data. This is particularly noteworthy given that the NESIL is intended to represent a dose at which sensitization would not be expected, while the ED_01_ allows for a 1 % chance across a HPPT population.

SARA-ICE PODs were also compared against reference EC3s (vertical green lines, Fig. 1). As expected, median ED_01_ estimates computed using only LLNA inputs into SARA-ICE are consistent with the reference EC3s. However, there is uncertainty when extrapolating from murine potency to human potency, and this is reflected in the widths of the 90 % credible intervals used to represent the estimate of the ED_01_. The SPUR for LLNA-only predictions ranges from 25 to 31, which is approximately 2-fold smaller than the largest difference between IT NESILs and reference EC3s – just less than 60-fold for BIT. Median ED_01_ estimates generated using only *in chemico/in vitro* inputs are closer to the NESIL than the reference EC3 for 4 out of the 5 possible comparisons.

We compared the SARA-ICE PODs against the EPA PODs generated as part of an IT case study (vertical blue lines, Fig. 1). The EPA PODs were derived from ANN model outputs from data generated by the DPRA, KS, and h-CLAT, and these are also utilized as input data in SARA-ICE (Table 3, Table 1 Supplemental) (Strickland et al., 2022, EPA, 2020). The ED_01_ was lower than the ANN output converted to a dose per skin area, by a factor of 4.6- to 65-fold. The correlation between the median ED_01_ estimate and the derived EPA POD is noticeably higher when the ED_01_ is estimated using *in chemico/in vitro* only, which is expected given the overlap in input data. The 5th percentile of the ED_01_ estimate is conservative relative to the EPA POD in nearly all comparisons.

Comparison of SARA-ICE results to GHS categories.

In chemical safety assessments, GHS categories can be utilized to help make decisions on personal protective equipment and quickly provide assessments of severity. Of the six IT compounds evaluated here, all but BIT and BBIT are GHS category 1A for skin sensitization according to ECHA. BIT and BBIT, while not able to be sub-categorized based on the existing data in ECHA, are considered GHS category 1 for hazard (Table 3). In order to classify a chemical into a GHS category, SARA-ICE produces probabilities of GHS classifications for both binary (1/NC) and sub-categorization (1A/1B/NC), which provides a level of confidence in the classification. Probabilities that are closer to 1 are higher confidence and must exceed pre-chosen confidence thresholds for both binary and subcategory classification. The example thresholds of $\theta_{\text{bin}}=0.77$ and $\theta_{\text{sub}}=0.62$ (see above section on GHS classification performance) are chosen to produce GHS classifications for the ITs. With these thresholds, the SARA-ICE GHS decision model returns a classification of “Sensitizer” (GHS Category 1) for all input combinations, with GHS classification probabilities for binary class 1 close to one for all sets of inputs (see Table 7). Classification probabilities for subcategory 1A are greater than 0.9 for all chemicals when estimated using *in chemico/in vitro* inputs only. These probabilities result in a GHS classification of 1A for all chemicals. MIT and BIT were both also classified as GHS 1A by the integrated testing strategy DA within OECD TG 497 (Annex 2) (OECD, 2021, OECD, 2021).

**Table 7:** GHS classification probabilities and classifications obtained from the SARA-ICE DA.

**Chemical**	**Input combination**	**Pr(GHS 1A)**	**Pr(GHS 1B)**	**Pr(NC)**	**Classification GHS binary**	**Classification GHS subcategory**
MIT	DPRA, KeratinoSens, h-CLAT	0.94	0.06	0.00	1	1A
MIT	LLNA	0.53	0.46	0.01	1	Inconclusive*
MIT	HPPT	0.54	0.45	0.00	1	Inconclusive*
MIT	DPRA, KeratinoSens, h-CLAT, LLNA	0.89	0.11	0.00	1	1A
MIT	DPRA, KeratinoSens, h-CLAT, HPPT	0.84	0.16	0.00	1	1A
MIT	HPPT, LLNA	0.61	0.39	0.00	1	Inconclusive*
MIT	DPRA, KeratinoSens, h-CLAT, HPPT, LLNA	0.86	0.14	0.00	1	1A
CMIT/MIT	DPRA, KeratinoSens, h-CLAT	0.98	0.02	0.00	1	1A
CMIT/MIT	LLNA	0.98	0.02	0.00	1	1A
CMIT/MIT	HPPT	1.00	0.00	0.00	1	1A
CMIT/MIT	DPRA, KeratinoSens, h-CLAT, LLNA	1.00	0.00	0.00	1	1A
CMIT/MIT	DPRA, KeratinoSens, h-CLAT, HPPT	1.00	0.00	0.00	1	1A
CMIT/MIT	HPPT, LLNA	1.00	0.00	0.00	1	1A
CMIT/MIT	DPRA, KeratinoSens, h-CLAT, HPPT, LLNA	1.00	0.00	0.00	1	1A
BIT	DPRA, KeratinoSens, h-CLAT	0.96	0.04	0.00	1	1A
BIT	LLNA	0.33	0.64	0.03	1	Inconclusive*
BIT	HPPT	0.83	0.16	0.00	1	1A
BIT	DPRA, KeratinoSens, h-CLAT, LLNA	0.81	0.19	0.00	1	1A
BIT	DPRA, KeratinoSens, h-CLAT, HPPT	0.98	0.02	0.00	1	1A
BIT	HPPT, LLNA	0.69	0.31	0.00	1	1A
BIT	DPRA, KeratinoSens, h-CLAT, HPPT, LLNA	0.93	0.07	0.00	1	1A
OIT	DPRA, KeratinoSens, h-CLAT	0.99	0.01	0.00	1	1A
OIT	LLNA	0.78	0.22	0.00	1	1A
OIT	DPRA, KeratinoSens, h-CLAT, LLNA	0.98	0.02	0.00	1	1A
DCOIT	DPRA, KeratinoSens, h-CLAT	0.99	0.01	0.00	1	1A
DCOIT	LLNA	0.98	0.02	0.00	1	1A
DCOIT	DPRA, KeratinoSens, h-CLAT, LLNA	1.00	0.00	0.00	1	1A
BBIT	DPRA, KeratinoSens, h-CLAT	0.97	0.03	0.00	1	1A

*The GHS sub-classification was inconclusive; the overall GHS classification is 1 for these chemicals. See supplementary material for more information and justification details. GHS classification cut-offs are set at a probability of 0.77 for 1/NC and 0.62 for 1A/1B sub-categorization.

Incorporating LLNA data into the estimate decreased the certainty of the classifications. For BIT, which has seven LLNA inputs with EC3s ranging from 1.5 % to 32 %, the subcategory classification is inconclusive, however the HPPT-only classification is 1A. The subcategory classification for MIT, using four LLNA inputs, is inconclusive, as is the HPPT-only classification and the combined HPPT and LLNA classification. The LLNA inputs for MIT have EC3s of 0.4 %, 0.86 %, 2.2 % and > 4.5 %. Converting to µg cm^−2^, this implies two EC3s less than the 500 µg cm^−2^ threshold for 1A/1B classification, and two greater. For BIT, five of the seven LLNA EC3s are greater than the 1A/1B threshold. For OIT, DCOIT, and CMIT/MIT, the difference in certainty between estimates with only *in vitro* input data, and the effect of addition of LLNA data was negligible, where the subcategory classification is also 1A when using LLNA data only. Estimated classification probabilities when combining all available *in chemico/in vitro* and LLNA inputs result in a GHS subcategory classification of 1A for all chemicals with an LLNA input.

Using DAs for assessing skin sensitization hazard, GHS categorization, and deriving quantitative values for risk assessment are preferred over animal methods as they are more predictive, human-relevant, efficient, reproducible, and cost-effective (OECD, 2021). Methods currently incorporated into OECD TG 497 do not derive PODs; they provide a single output (hazard or GHS classification) with no flexibility in the model output. SARA-ICE is currently under evaluation at the OECD for inclusion in the OECD TG 497, as a model for derivation of PODs for risk assessment. An advantage of the SARA-ICE DA is that, as a probabilistic Bayesian model, it can use partial information sources to derive the ED_01_, and also has flexibility in the output, from hazard classification through to the derivation of a POD, with a measure of certainty around each output. Within the derivation of the ED_01_, there is additional flexibility to determine where in the distribution a value should be used, even within the confines of a defined data interpretation procedure, such as the 5th percentile for a more conservative estimate, or for threshold cut-offs for GHS classification. In the case of the ITs, the ED_01_ is comparable or more conservative than expert judgement-based, weight-of-evidence derived values such as NESILs. DAs like SARA-ICE are advantageous for multiple reasons – primarily in that it derives human-relevant skin sensitization potential and potency estimates, without human or animal models. Additionally, it allows for partial/variable data inputs, and it can be rapidly applied to predict a POD for skin sensitization risk assessment. Concurrent to the efforts at OECD, the SARA-ICE model will be made publicly available as a locally downloadable package and hosted on the ICE website for easy access to all users.

## Conclusions

The SARA-ICE DA represents an important advancement in the field of risk assessment for skin sensitization by combining NAMs and computational approaches to improve public health protection. Using a case study on a group of known, widespread sensitizing substances, we have demonstrated that SARA-ICE provides robust and reliable results for PODs to be used in skin sensitization risk assessments. The model has been shown to perform well using reference data from the TG 497 Annex 2 (OECD, 2021), with most performance metrics exceeding 80 % for hazard and GHS potency estimates. It is noted that with the evaluated thresholds ($\theta_{\text{bin}}$ = 0.77 and $\theta_{\text{sub}}$ *=* 0.62) the inconclusive rate does approach 50 % for some categories, which should be taken into account when selecting the required thresholds. With lower probability thresholds, the inconclusive rate does decrease.

Trained on the largest available curated skin sensitization reference database, the probabilistic Bayesian model SARA-ICE provides quantitative potency estimates with associated prediction intervals, using a variety of input data types. When used as model inputs, human biology-based NAMs that represent key molecular and cellular processes provide more confident and human health-protective PODs. This makes the SARA-ICE DA consistent with guidance from the OECD on using DAs for hazard and potency classification.

## Funding

This project was partially funded with federal funds from the National Institute of Environmental Health Sciences, National Institutes of Health under Contract No. HHSN273201500010C to Inotiv-RTP and by National Institutes of Health Intramural Research Project ES103386-01, Research Operations Supporting the National Toxicology Program Interagency Center for the Evaluation of Alternative Toxicological Methods.

## Declaration of competing interest

The authors declare that they have no known competing financial interests or personal relationships that could have appeared to influence the work reported in this paper.

## Footnotes

3 Here, $\text{logit}\left(x\right)=\text{log}\left(\frac{x}{1-x}\right)$ using the natural base.
