# Supplementary material for: The skin allergy risk assessment-integrated chemical environment (SARA-ICE) defined approach to derive points of departure for skin sensitization
Source: Curr Res Toxicol. 2024 Dec 14;8:100205. doi: 10.1016/j.crtox.2024.100205 (PMC11719337; doi:10.1016/j.crtox.2024.100205)
Supplement: Supplementary Data 1 [file mmc1.docx]

SKIN ALLERGY RISK ASSESSMENT (SARA) – INTEGRATED CHEMICAL ENVIRONMENT (ICE) DEFINED APPROACH (DA)

# Acronyms

SARA – skin allergy risk assessment

ICE – integrated chemical environment

DA – defined approach

NICEATM – NTP Interagency Center for the Evaluation of Alternative Toxicological Methods

AOP – adverse outcome pathway

HPPT – human predictive patch test

HRIPT – human repeat insult patch test

HMT – human maximisation test

LLNA – local lymph node assay

DPRA – direct peptide reactivity assay

CDF – cumulative density function

OECD – Organisation for Economic Co-operation and Development

# Summary

The SARA-ICE DA employs a Bayesian statistical model to describe the relationships between, and variability within data from seven study types including the LLNA, (OECD, 2010), HPPT (either HRIPT (Politano & Api, 2008) or HMT (Kligman, 1966; Kligman & Epstein, 1975)), DPRA and kDPRA (OECD, 2020), h-CLAT (OECD, 2018b), KeratinoSens^TM^ (OECD, 2018a) and U-Sens^TM^ (OECD, 2018b). Various combinations of data from these study types may be used as inputs to the model. The primary outputs of the SARA-ICE DA are: a) a human-relevant continuous metric of sensitiser potency and b) a UN Globally Harmonized System of Classification and Labelling of Chemicals (GHS) classification (UN, 2021). Both metrics are estimated in the form of a probability distribution reflecting the uncertainty in the estimate.

The SARA-ICE DA metric of sensitiser potency is defined as the dermal dose at which there is a 1% sensitisation rate within a HPPT-eligible population. This is denoted as the ED_01_. The SARA-ICE DA builds on the previously published SARA models in (Reynolds et al., 2022; Reynolds, MacKay, Gilmour, Miguel-Vilumbrales, & Maxwell, 2019). The SARA-ICE DA can be used to obtain sensitiser potency estimates and UN GHS classifications from *in vitro* data only. However, the model may also estimate these quantities using *in vivo* data (HPPT, LLNA), or combinations of both *in vivo* and *in vitro* data for a weight-of-evidence estimate.

# The SARA-ICE database

## Input types

The SARA-ICE DA utilises data from the following sources:

1. Human patch test data; HPPT (Human Repeat Insult Patch Test (HRIPT) (Politano & Api, 2008) and human maximization test (HMT (Kligman, 1966; Kligman & Epstein, 1975)). Only studies in which the cohort size, number sensitised and applied dose are reported are admissible as data into the approach.
2. Local Lymph Node Assay (LLNA, OECD TG 429). EC_3_ values obtained from individual studies are admissible data. Representative or averaged values are not admissible.
3. Direct Peptide Reactivity Assay (DPRA, OECD TG 442c). Maximum percentage depletion with either cysteine and/or lysine peptides (%).
4. Kinetic DPRA (OECD TG 442c). log Kmax value (typical units of M^-1^s^-1^ must be converted to gL^-1^s^-1^).
5. KeratinoSens^TM^ Assay (OECD TG 442D). Reported EC_1.5_ value (typical units of µM must be converted to µg ml^-1^).
6. h-CLAT Assay (OECD TG 442E). Reported CD54 EC_200_, CD86 EC_150_ and CV_75_ (units µg ml^-1^).
7. U-Sens^TM^ Assay (OECD TG 442E). Reported CD86 EC_150_ (units µg ml^-1^).

The SARA-ICE DA may be run with subsets of these information sources, e.g. with *in vitro* assay data only.

## Data curation

The curated SARA-ICE database consists of 434 distinct chemicals, identified using CASRN. The number of study results per study type tallies at 871 HPPT (365 HRIPT, 506 HMT), 536 LLNA, 650 DPRA, 361 kDPRA, 972 KeratinoSens^TM^, 431 h-CLAT and 164 U-Sens^TM^. These are divided heterogeneously between each CASRN, with the minimum number of studies per chemical equal to two.

HPPT data were curated in accordance with the OECD Guideline 497 project (see Annex IV in (OECD, 2021b)). Briefly, the documentation of each test was reviewed to determine protocol (HMT or HRIPT), protocol subtype, identification of the test substance, applied dose, number of subjects tested, and number of subjects responding. Because they were assumed to be significantly different from the standard tests, tests with an unspecified subtype, “other,” were excluded unless they had been used in published safety assessments from the Research Institute for Fragrance Materials (RIFM) or the Scientific Committee on Consumer Safety (SCCS). Additional exclusions included tests without a dermal surface area (DSA) value, tests for which subject numbers or doses were reported as ranges, and tests of substances of variable or ill-defined composition, such as natural extracts and other mixtures.

LLNA data were also curated in accordance with the OECD Guideline 497 project (see Annex III in (OECD, 2021b)). LLNA studies with three or four days of exposure to topical application of test substance on both ears of the mice were accepted. Acceptable tests also used a radiolabelled marker and measured lymphocyte proliferation in the lymph nodes draining the site of test substance application during the induction phase of skin sensitisation in either pooled or individual animals. Tests must include a vehicle control and reports must provide all test concentrations and corresponding stimulation index (SI) values. Tests were excluded if sodium lauryl sulfate was applied to enhance the response or if the EC_3_ values were extrapolated and did not meet the following criteria (Ryan et al., 2007):

- The lowest measured SI value was < 5
- The extrapolated EC_3_ was less than 10-fold of the closest tested concentration
- The slope ratio was ≤ 2 and non-negative. This value is the ratio of the slope from the high dose to the mid-dose, to the slope from the mid-dose to the lowest dose.

NAM data (DPRA, KeratinoSens^TM^, h-CLAT and U-Sens^TM^) were first obtained from the ICE database. Additional studies were obtained from (Hoffmann et al., 2022) and (Reynolds et al., 2022). Compiled databases were checked for duplicate studies via manual inspection of study results and references.

Once all data had been sourced, chemicals with a single study in total across all study types were removed. The computational burden of using the SARA-ICE model scales with the number of chemicals in the core SARA-ICE database. The primary purpose of the core SARA-ICE database is to estimate chemical-agnostic model variables. Chemicals with a single study in total across all study types provide no information towards learning chemical-agnostic model variables, therefore were removed for computational efficiency.

## Assay results and conversion to observations of SARA-ICE variables

The SARA-ICE model describes, probabilistically, relationships between data from HPPTs, LLNAs and *in vitro* assays including the DPRA, kDPRA, KeratinoSens^TM^, h-CLAT and U-Sens^TM^. Prior to use within the model, data from each of the study types listed in Section 6 undergo a deterministic transformation. The purpose of this transformation is to: a) convert data points to a unit scale for computational stability, and b) ensure it is reasonable to assume linear relationships between model variables corresponding to each study type. Details of the applied transformations are provided below.

### HPPT

The information taken from a HPPT study includes the dermal induction dose (µg cm^-2^), the total number of test subjects and the total number of subjects sensitised following challenge. The dermal induction dose is converted to its base-10 logarithm before use in the model.

### LLNA

The information taken from an LLNA is the EC_3_. The EC_3_ is first converted to units of µg cm^-2^ and then the base-10 logarithm is calculated. For negative LLNAs in which an EC_3_ is unavailable, the input is $>x$ where $x$ is the base-10 logarithm of the maximum tested concentration in units of µg cm^-2^. The core SARA-ICE database contains EC_3_s calculated via extrapolation to a value less than the minimum concentration tested. However, in instances where this cannot be done reliably the SARA-ICE input may be provided as $<x$ where $x$ is the base-10 logarithm of the minimum concentration tested.

### DPRA

From the DPRA, we determine the maximum depletion of either the cysteine or lysine peptides. Assuming the maximum depletion is expressed as a percentage $y$, and the value is between 1% and 99%, this is converted to the value

$$x=\text{log}\left( \frac{100\%-y}{y} \right) ,$$

where the base of the logarithm is Euler’s constant. This conversion is essentially the logit transformation and serves to map the percentage depletion to a real number.

If the maximum depletion is less than 1%, which includes studies in which the reported maximum percentage depletion is negative, then the percentage depletion is expressed as <1%. In such cases, the input to SARA-ICE is $>x_{\text{max}}$, where

$$x_{\text{max}}=\text{log}\left( \frac{100\%-1\%}{1\%} \right)\approx4.595 .$$

Alternatively, if the maximum depletion is greater than 99%, the input to SARA-ICE is $<x_{\text{min}}$ where

$$x_{\text{min}}=\text{log}\left( \frac{100\%-99\%}{99\%} \right)\approx-4.595 .$$

### Kinetic DPRA

To use the kinetic DPRA as an input type, the log Kmax value is first converted to have units g L^-1^ s^-1^. This is readily achieved by subtracting the base-10 logarithm of the chemical’s molecular weight from the log Kmax value when in typical units of M^-1^ s^-1^. For input into SARA-ICE, the converted log Kmax value is multiplied by -1. This transformation is performed to ensure a positive correlation between the input and the ED_01_, i.e., low values of the input correspond to low ED_01_ values.

### KeratinoSens^TM^

The information used within SARA-ICE from a KeratinoSens^TM^ assay is the reported EC_1.5_ value and the IC_50_. These values are first converted to units of µg ml^-1^ following which the base-10 logarithm of each is calculated. Since the usual reported units for a KeratinoSens^TM^ assay are µM, it is usually necessary to know the molecular weight of a substance to use a KeratinoSens^TM^ result in the SARA-ICE DA.

If the KeratinoSens^TM^ assay exhibits no concentration-response, e.g., if the luciferase expression is less than 1.5-fold greater than control at all test concentrations, then the input into SARA-ICE is $>x$ where $x$ is the maximum concentration tested within the assay and the conversion above is similarly followed. The SARA-ICE model may also accept inputs of the form $<x$ which may occur if there was more than 1.5-fold increase in luciferase activity at all tested concentrations.

### h-CLAT

The information taken from the h-CLAT assay is the minimum of either reported CD86 EC_150_ or CD54 EC_200_, and the CV_75_ value. If only one of CD86 EC_150_ and CD54 EC_200_ is reported, the reported value is used. If neither value is reported, the input is $>x$ where $x$ is the maximum concentration tested. This is inferred as 1.2 times the CV_75_ if not provided explicitly. Units of inputs are assumed to be µg ml^-1^ and base-10 logarithms are calculated. If either of the CD86 EC_150_ or CD54 EC_200_ are reported as $<x$, this also forms a valid input.

### U-Sens^TM^

The information taken from the U-Sens^TM^ assay is the CD86 EC_150_ and CV_70_ in units of µg ml^-1^. Similarly to above, both inputs are transformed to their base-10 logarithm. If the U-Sens^TM^ study is negative, the input is $>x$ where $x$ is the maximum concentration tested. The input $<x$ may also be supplied, for example, if the CD86 EC_150_ was determined to be lower than the minimum concentration tested.

# The SARA-ICE model

In Bayesian statistics, there is no distinction between model variables and data (a data point is simply an observation of one of the random variables within the model). In the case of the SARA-ICE DA, the number of variables within the model scales with the number of chemicals in the database. There are 21 variables reserved for each chemical, which for the core database results in a total of 21 x 434 = 9,114 chemical-specific variables. Three chemical-specific variables (one location-like, two scale-like) are reserved for the HPPT, whilst two variables (one location-like and one scale-like) are reserved for each of the other input types. Location-like variables are used to describe the first moment (average) of some quantity whilst scale-like variables are used to describe the second moment (variance) of the same distribution. The HPPT has two scale-like chemical-specific variables, one for population variability in individual thresholds for sensitisation and the second for variability in HPPT test conditions.

The SARA-ICE model also includes a far smaller number of “chemical-agnostic” variables. These variables are not specific to any chemical and form the core set of variables of the model. All of these variables are present in the model irrespective of the number of chemicals. A subset of these describes the relationships between the chemical-specific location-like variables. A second subset are used to describe how chemical-specific scale-like parameters vary across chemical space. In what follows, the subscript $i$ is used to index a chemical within the model. If a variable is written with subscript $i$, this implies the variable is chemical-specific. Alternatively, if a variable is written without subscript $i$, it can be assumed to be chemical-agnostic.

## Modelling HPPT data

The primary variable of interest within the SARA-ICE DA is the chemical-specific random variable $\theta_{i}$, defined as the base-10 logarithm of the HPPT dose expected to sensitise 1% of a HPPT-eligible population (ED_01_). Within the SARA-ICE model, both HRIPT and HMT studies are treated identically. Each $\theta_{i}$ is assigned the prior distribution

$$\theta_{i}\sim\text{Normal}\left( \theta_{\text{loc}},\theta_{\text{scale}} \right) ,$$

with $\theta_{\text{loc}}=3.738$ and $\theta_{\text{scale}}=2.413$. Prior distribution parameters are chosen such that within the prior

$$\text{Pr}\left( \theta_{i}\leq\text{lo}\text{g}_{10}\left( 500 \right) \right)\approx\frac{1}{3} ,$$

$$\text{Pr}\left( \theta_{i}\geq\text{lo}\text{g}_{10}\left( 60,000 \right) \right)\approx\frac{1}{3} .$$

The motivation for this choice is discussed in the GHS section below.

An individual from a HPPT-eligible population (indexed by $j$), is assumed to possess a personal threshold for sensitisation to chemical $i$, denoted $\hat{\theta}_{i,j}$, such that, for study dose $d$, if $d<\hat{\theta}_{i,j}$, the individual does not present with contact allergy upon challenge and if $d\geq\hat{\theta}_{i,j}$, then the individual will be counted as sensitised. The distribution of thresholds within a population is assumed to be normally distribution on the logarithmic scale and the probability density function for this is re-parameterised in terms of the 1^st^ percentile ($\theta_{i}$), rather than the mean. The cumulative density function for this distribution of personal thresholds for induction of sensitisation to chemical $i$ is expressed as

$$\text{Pr}\left( \hat{\theta}_{i,j}\leq x \mid\theta_{i},s_{i} \right)=F\left( x \mid\theta_{i},s_{i} \right)=\Phi\left( \frac{x-\theta_{i}}{s_{i}}+\Phi^{-1}\left( 0.01 \right) \right) ,$$

where $\Phi\left( \cdot\right)$ is the cumulative density function (CDF) of a standard normal distribution. Whilst individual thresholds for sensitisation may vary in time, the overall distribution is assumed stationary with respect to time. The variable $s_{i}$ gives the standard deviation of individual thresholds for induction of sensitisation (on the log-10 scale). Estimates of this variable are regularised using the prior structure

$$s_{i}\sim\text{Weibull}\left( 5,s_{\text{scale}} \right) ,$$

$$s_{\text{scale}}\sim\text{HalfNormal}\left( 0,1 \right) .$$

The probability of sensitisation within a HPPT study (indexed by $k$) is assumed to vary in response to experimental choices such as the vehicle used to apply the chemical. Given the nominal study induction dose $d_{i,k}$ for chemical $i$, the probability of sensitisation at $d_{i,k}$ is modelled as

$$p_{i,k}=\text{logi}\text{t}^{\text{-1}}\left( \text{logit}\left( \Phi\left( \frac{d_{i,k}-\theta_{i}}{s_{i}}+\Phi^{-1}\left( 0.01 \right) \right) \right)+\lambda_{i,k} \right)$$

where $\lambda_{i,k}$ is a study-specific offset (on the logit scale) such that study-to-study variability in results can be adequately accounted for. Estimates of $\lambda_{i,k}$ are regularised with the prior structure

$$\lambda_{i,k}\sim\text{Weibull}\left( 5,\lambda_{\text{scale}} \right) ,$$

$$\lambda_{\text{scale}}\sim\text{HalfNormal}\left( 0,2 \right) .$$

Given probability of sensitisation $p_{i,k}$, the number of subjects sensitised, $n_{i,k}$ out of those tested $N_{i,k}$ is assumed to be binomially distributed such that

### Numerical stability when calculating the normal distribution CDF

The use of the CDF of a standard normal distribution may result in numerical instability when performing floating point arithmetic. To counter this, we use the analytical approximation to the standard normal CDF provided in the documentation for the Stan programming language (Carpenter et al., 2017). For numerical stability, we let

$$\Phi\left( x \right)\approx\text{logi}\text{t}^{\text{-1}}\left( 0.07056x^{3}+1.5976x \right) .$$

## Modelling correlations between study types

Each chemical in the model is assigned ten location-like variables, one of which is $\theta_{i}$ as introduced in the previous section. The other nine variables describe the (latent) mean for chemical $i$ in terms of each of the other inputs. These include the LLNA mean EC_3_, DPRA mean maximum depletion, kinetic DPRA mean reactivity rate, KeratinoSens^TM^ mean EC_1.5_ and mean IC_50_, h-CLAT mean minimum of the CD86 EC_150_ and CD54 EC_200_ biomarkers and mean CV_75_, and U-Sens^TM^ mean CD86 EC_150_ and mean CV_70_. As a more specific example, considering the input transformations defined above, let $\bar{y}_{i,\text{L}}$ be the location-like variable for chemical $i$ in the LLNA. Then, after reversing the transformation defined above, ${10}^{\bar{y}_{i,\text{L}}}$ is the average EC_3_ (µg cm^-2^) for chemical $i$ in the LLNA.

We use the subscript $s\in S=\left\{ \text{L},\text{D},\text{kD},\text{K},\text{Kc},\text{H},\text{Hc},\text{U},\text{Uc} \right\}$ to distinguish the nine location-like variables $\bar{y}_{i,s}$ for chemical $i$ for each input type. The vector $\bar{y}_{i}$ is composed from the collection of all $\bar{y}_{i,s}$ for the non-HPPT input types. The role of the study result conversions defined above is to ensure it is reasonable to assume each $\bar{y}_{i,s}$ is linearly associated with $\theta_{i}$. Conditional on $\theta_{i}$, it is assumed that

$$\bar{y}_{i}\sim\text{MultivariateNormal}\left( \boldsymbol{\alpha}+\boldsymbol{\beta}\left( \theta_{i}-\theta_{\text{loc}} \right),\Sigma\right) ,$$

where $\boldsymbol{\alpha}=\left[ \alpha_{\text{L}},\ldots,\alpha_{\text{Uc}} \right]$ and $\boldsymbol{\beta}=\left[ \beta_{\text{L}},\ldots,\beta_{\text{Uc}} \right]$ are vectors of intercept and slope variables, respectively. The covariance matrix $\Sigma$ is defined as

$$\Sigma=\text{diag}\left( \gamma_{\text{L}},\ldots,\gamma_{\text{Uc}} \right) \Omega\text{diag}\left( \gamma_{\text{L}},\ldots,\gamma_{\text{Uc}} \right)^{T}$$

where $\gamma_{s}$, $s\in S$ are scale parameters defining the marginal standard deviations of the regression residuals and $\Omega$ is an $9\times9$ correlation matrix with elements $\rho_{s,t}$ for all possible pairs $s\in S$, $t\in S$. Each scale parameter is assigned the prior distribution

$$\gamma_{s}\sim\text{HalfNormal}\left( 0,2 \right)$$

and the correlation matrix $\Omega$ is assigned a Lewandowski-Kurowicka-Joe prior distribution with shape parameter equal to 2 (Lewandowski, Kurowicka, & Joe, 2009):

$$\Omega\sim\text{LKJ}\left( 2 \right) .$$

Prior distributions for the intercept and slope parameters are as follows:

$$\alpha_{\text{L}}\sim\text{Normal}\left( 3,1 \right) ,$$

$$\alpha_{\text{D}}\sim\text{Normal}\left( 0,2 \right) ,$$

$$\alpha_{\text{kD}}\sim\text{Normal}\left( 5,2 \right) ,$$

$$\alpha_{s}\sim\text{Normal}\left( 2,1 \right)\quad\text{for }s\in\left\{ \text{K},\ldots,\text{Uc} \right\} ,$$

$$\beta_{\text{L}}=1\quad\left( \text{This variable is fixed at a constant} \right) ,$$

$$\beta_{s}\sim\text{Normal}\left( 2,1 \right)\quad\text{for} s\in\left\{ \text{D},\text{kD} \right\} ,$$

$$\beta_{s}\sim\text{Normal}\left( 1,1 \right)\quad\text{for} s\in\left\{ \text{K},\ldots,\text{Uc} \right\} .$$

Notice that the LLNA slope variable is fixed to a constant equal to one. This enforces the assumption that murine sensitiser potency is proportional to human sensitiser potency (when expressed on a linear axis).

## Modelling input variability

Given location-like variables $\bar{y}_{i,s}$, converted study results $y_{i,j,s}$(where $j$ indexes repeat studies) are assumed to be normally distributed such that

$$y_{i,j,s}\sim\text{Normal}\left( \bar{y}_{i,s},\sigma_{i,s} \right)$$

where $\sigma_{i,s}$ is the standard deviation of converted study results for chemical $i$ for input type $s\in S$. These variables are regularised using the prior structure

$$\sigma_{i,s}\sim\text{Weibull}\left( 5,\sigma_{\text{scale},s} \right) ,$$

$$\sigma_{\text{scale},s}\sim\text{HalfNormal}\left( 0,1 \right)\quad\text{for} s\in S .$$

The idea here is that each input variability is distinct for different chemicals and input types. The Weibull prior serves to shrink variability parameters estimates towards each other. In the event that a chemical has no repeat studies for a particular assay, the estimate of the $\sigma_{i,s}$ will be distributed as its prior distribution $\text{Weibull}\left( 5,\sigma_{\text{scale},s} \right)$.

### Censored data

In the event that an assay has a negative result, the input into the SARA-ICE model may be expressed as an inequality (< or >). These are examples of *censored data*. In such cases we integrate over the prior distribution for this observation from the censoring point. For example, if the converted datapoint is $>y_{\text{obs}}$, then the probability of this event occurring is

$$\text{Pr}\left( y_{i,j,s}>y_{\text{obs}}\mid\bar{y}_{i,s},\sigma_{i,s} \right)=1-\text{Pr}\left( y_{i,j,s}\leq y_{\text{obs}}\mid\bar{y}_{i,s},\sigma_{i,s} \right)=1-\Phi\left( \frac{y_{\text{obs}}-\bar{y}_{i,s}}{\sigma_{i,s}} \right) .$$

Similarly, if the converted datapoint is $<y_{\text{obs}}$, then

$$\text{Pr}\left( y_{i,j,s}<y_{\text{obs}}\mid\bar{y}_{i,s},\sigma_{i,s} \right)=\text{Pr}\left( y_{i,j,s}\leq y_{\text{obs}}\mid\bar{y}_{i,s},\sigma_{i,s} \right)=\Phi\left( \frac{y_{\text{obs}}-\bar{y}_{i,s}}{\sigma_{i,s}} \right) .$$

The use of the standard Gaussian CDF may result in numerical instability. Therefore, we use the approximation recommended in the Stan programming language technical manuals (see section on computational approximation above).

## Inferences from the SARA-ICE model

The SARA-ICE model defined in the previous section describes the probability distribution of (transformed) observations from the HPPT, LLNA, DPRA, kDPRA, KeratinoSens^TM^, h-CLAT and U-Sens^TM^ conditional on the assumed prior structure. Let $\Theta$ be the joint distribution of all model variables and $\mathcal{D}$ be the joint distribution of all data. Then the previous section defines the density

$$p\left( \mathcal{D} \mid\Theta\right) p\left( \Theta\right) .$$

Bayes theorem defines the conditional probability,

$$p\left( \Theta\mid\mathcal{D} \right)=\frac{p\left( \mathcal{D} \mid\Theta\right) p\left( \Theta\right)}{p\left( \mathcal{D} \right)} ,$$

the left side of which expresses the joint probability distribution of all model variables, conditional on a set of observations $\mathcal{D}$, such as those in the core SARA-ICE database. Estimates of quantities of interest, such as the HPPT 1% sensitising dose for a particular chemical with data in $\mathcal{D}$, are obtainable as marginal distributions of $p\left( \Theta\mid\mathcal{D} \right)$.

### Computation

The distribution $p\left( \Theta\mid\mathcal{D} \right)$ is analytically intractable (cannot be evaluated directly) therefore it is evaluated by drawing samples of model parameters from it. Sampling is achieved using Monte Carlo Markov Chain (MCMC) approaches. Whilst any state-of-the-art sampler may be used for this purpose, the Python API to the programming software Stan (Carpenter et al., 2017) has been used to realise the model in a computational environment. The number of draws from the posterior distribution is typically chosen to be 10,000, obtained by running 20 chains of 1,000 iterations each and discarding the first 500 samples as burn-in.

Samples of model variables may be used to estimate the marginal densities of variables of interest (or even transformations of model variables of interest). For example, the density of the ED_01_ for some chemical of interest can be visualised in the form of a histogram using samples ${10}^{\theta_{i,k}}$ where $\theta_{i,k}$ is the $k$th draw of variable $\theta_{i}$. Other quantities of interest such as posterior expectations, variances etc. can be obtained by computing sample means and variances from the set of draws of model variables. Using the current SARA-ICE database and model as a benchmark, the computational time using a 24-core CPU is around one hour to obtain 10,000 samples from the posterior distribution.

It is important to note that MCMC techniques which are used to obtain parameter estimates are probabilistic methods. This implies estimates have a degree of variation to them. The magnitude of the variation can be controlled by adjusting by the number of draws, but this comes at the cost of an increased computational load. The default choice of 10,000 draws is usually sufficient to ensure posterior quantities, such as mean estimates, are reproducible to two significant figures. As a defined approach, this implies that SARA-ICE DA inferences will not produce identical outputs even if the DA is run on the same dataset multiple times (unless random number generators used are initialised with the same seed). The minor variance in SARA-ICE DA estimates is judged to represent an acceptable level of reproducibility for regulatory use (e.g. if benchmarked against the level of variance in experimental results from the assays used to inform SARA-ICE predictions), however, it could be further reduced with increased computation time, if needed.

### Inferences for chemicals not in the core SARA-ICE database

Suppose we are interested in obtaining inferences for a chemical not in the SARA-ICE database, but for which we have some assay results. To obtain inferences for this chemical, its data must be merged with SARA-ICE database to create a new dataset $\tilde{\mathcal{D}}$ following which we evaluate the distribution $p\left( \Theta,\Theta_{\text{chemical}} \mid\tilde{\mathcal{D}} \right)$, where $\Theta_{\text{chemical}}$ are the additional chemical-specific model variables. The joint distribution of chemical-specific variables can be obtained as

$$p\left( \Theta_{\text{chemical}} \mid\tilde{\mathcal{D}} \right)=\int_{\Theta} p\left( \Theta,\Theta_{\text{chemical}} \mid\tilde{\mathcal{D}} \right) \text{d}\Theta.$$

The posterior distribution in this case needs to be evaluated using the computational techniques discussed above. Because the full SARA-ICE database, plus some extra datapoints are being used, the computational time sample from the posterior distribution is roughly the same at 1 hour using a 24-core CPU. This can be prohibitively slow if deploying the model within a production environment. An approach to address this is provided in the following section.

## Ensuring rapidly obtainable results for a chemical not in the core SARA-ICE database

Given a small set of study results for some chemical of interest which is not in the core SARA-ICE database, we aim to implement a computational environment that enables estimation of the chemical-specific variables for this chemical in seconds, not hours.

This is achieved by first evaluating the posterior distribution $p\left( \Theta\mid\mathcal{D} \right)$ conditional on the core SARA-ICE database $\mathcal{D}$. The set of model variables $\Theta$ is split into those which are chemical-agnostic, $\Theta_{\text{agnostic}}$ and those which are chemical-specific, $\Theta_{\text{specific}}$. We set up an analytical approximation to the joint distribution of chemical-agnostic variables

$$p\left( \hat{\Theta} \right)\approx\int_{\Theta_{\text{specific}}} p\left( \Theta_{\text{specific}},\Theta_{\text{agnostic}} \mid\mathcal{D} \right) \text{d}\Theta_{\text{specific}}$$

and replace the original prior in the SARA-ICE with this approximation. Letting $\mathcal{D}_{\text{chemical}}$ be the data for the chemical of interest, the joint distribution of model variables for this chemical is approximated as

$$p\left( \Theta_{\text{chemical}}\mid\mathcal{D}_{\text{chemical}} \right)=\int_{\hat{\Theta}} \frac{p\left( \mathcal{D}_{\text{chemical}} \mid\Theta_{\text{chemical}} \right)p\left( \Theta_{\text{chemical}} \mid\hat{\Theta} \right)p\left( \hat{\Theta} \right)a}{p\left( \mathcal{D}_{\text{chemical}} \right)} \text{d}\hat{\Theta} .$$

MCMC techniques are used to evaluate this distribution, however, the number of data points is small, and correspondingly, the number of chemical-specific model variables is small. This computation can be performed in a matter of seconds as opposed to the hours when performed over the 1,000s of datapoints in the core SARA-ICE database.

This SARA-ICE model with the prior distribution replaced by an approximation of the marginal posterior conditioned on the SARA-ICE database is referred to as the SARA-ICE production model. The method of obtaining an analytical approximation to the full posterior is outlined in the next section.

### Analytical approximation of the SARA-ICE posterior distribution

The chemical-agnostic variables are split into two groups: the first group consists of all $\gamma_{s}$, $s\in S$ and $\rho_{s,t}$, $s,t\in S$ making up the covariance matrix $\Sigma$ defined above. The second group consists of everything else which includes $\alpha_{s}$, $\beta_{s}$ and $\sigma_{\text{scale},s}$ for $s\in S$ (except $\beta_{\text{L}}$ which is constant) and lastly $s_{\text{scale}}$ and $\lambda_{\text{scale}}$.

The marginal posterior distribution of $\Sigma$ is approximated using a Wishart distribution. The statistical model

$$\Sigma\mid\mathcal{D}\sim\text{Wishart}\left( \Psi,\nu\right) ,$$

$$\nu\propto1 ,$$

$$\Psi=\text{diag}\left( \omega\right)\Omega\text{diag}\left( \omega\right) ,$$

$$\omega\sim\text{HalfNormal}\left( 0,1 \right) ,$$

$$\Omega\sim\text{LKJ}\left( 2 \right) ,$$

is assumed and samples from the density $p\left( \Psi,\nu\mid\Sigma\mathcal{,D} \right)$ are obtained using Stan. The posterior expectations

$$\bar{\Psi}=\text{E}\left[ \Psi\mid\Sigma\mathcal{,D} \right] ,$$

$$\bar{\nu}=\text{E}\left[ \nu\mid\Sigma\mathcal{,D} \right]$$

are calculated from which we define an approximate sampling distribution for the posterior distribution of $\Sigma$ as

$$\Sigma\sim\text{Wishart}\left( \bar{\Psi},\bar{\nu} \right) .$$

The marginal distribution of the second group of variables is approximated using a multivariate Gaussian. Some of these random variables are strictly positive, therefore the multivariate Gaussian is used to approximate the sampling distribution for the logarithm of these variables. Define the vector

$$\boldsymbol{\theta}=\left[ \text{log}\left( s_{\text{scale}} \right),\text{log}\left( \lambda_{\text{scale}} \right),\alpha_{\text{L}},\ldots,\alpha_{\text{Uc}},\beta_{\text{D}},\ldots,\beta_{\text{Uc}},\text{log}\left( \sigma_{\text{scale},\text{L}} \right),\ldots,\text{log}\left( \sigma_{\text{scale},\text{L}} \right) \right]^{T} ,$$

then the parameters statistical model

$$\boldsymbol{\theta}\sim\text{MultivariateNormal}\left( \boldsymbol{\mu}_{\theta},\Sigma_{\theta} \right)$$

are estimated using maximum likelihood estimates ${\hat{\boldsymbol{\mu}}}_{\theta}$ and $\hat{\Sigma}_{\theta}$. These then define the approximation to the marginal posterior distribution of $\boldsymbol{\theta}$ as

$$\boldsymbol{\theta}\sim\text{MultivariateNormal}\left( {\hat{\boldsymbol{\mu}}}_{\theta},\hat{\Sigma}_{\theta} \right) .$$

The prior distribution of chemical agnostic parameters is then defined as the product

$$p\left( \hat{\Theta} \right)\propto p\left( \bar{\Psi},\bar{\nu} \right)p\left( {\hat{\boldsymbol{\mu}}}_{\theta},\hat{\Sigma}_{\theta} \right) .$$

This approximation will preserve correlations between variables within $\Sigma$ and between variables within $\boldsymbol{\theta}$, however, any correlations between these two are lost within the approximation. Practically, this loss of information results in small differences between ED_01_ estimates obtained when using this prior and only chemical-specific information.

## GHS classification

### GHS classification probabilities

The distribution for the ED_01_ is discretised into three intervals corresponding to GHS sensitiser categories. This is done by choosing a HPPT dermal threshold above which sensitiser potency is so low that the chemical can be assumed, for all intents and purposes, as non-sensitising. The chosen threshold is 60,000 µg cm^-2^, corresponding to the maximum achievable dose in a standard HPPT (concentration at 100%, assuming 0.3 g applied within a 25 mm Hill top chamber) (Politano & Api, 2008). The SARA-ICE probability that a chemical is a sensitiser is simply the probability that the ED_01_ is less than this threshold.

To represent this formally, let the discrete random variable $\mathcal{G}_{\text{bin},i}$ represent the SARA-ICE binary GHS category for the chemical $i$. The outcome space for this random variable is the set {1, NC}, the elements of which correspond to GHS binary class 1 and “not requiring classification” (equivalent to a classification of a chemical not being a sensitiser). Then we define

$$\text{Pr}\left( \mathcal{G}_{\text{bin},i}=1 \mid\theta_{i} \right)=\text{Pr}\left( \theta_{i}\leq\text{lo}\text{g}_{10}\left( 60,000 \right) \right) ,$$

$$\text{Pr}\left( \mathcal{G}_{\text{bin},i}=\text{NC} \mid\theta_{i} \right)=\text{Pr}\left( \theta_{i}>\text{lo}\text{g}_{10}\left( 60,000 \right) \right) .$$

A lower threshold of 500 µg cm^-2^ is used as a boundary between GHS subcategories 1A and 1B (OECD, 2021a). Let the second discrete random variable $\mathcal{G}_{\text{sub},i}$ represent the SARA-ICE subcategory GHS classification for chemical $i$. The outcome space for this random variable is the set {1A, 1B, NC} with probabilities

$$\text{Pr}\left( \mathcal{G}_{\text{sub},i}=\text{1A} \mid\theta_{i} \right)=\text{Pr}\left( \theta_{i}\leq\text{lo}\text{g}_{10}\left( 500 \right) \right) ,$$

$$\text{Pr}\left( \mathcal{G}_{\text{sub},i}=\text{1B} \mid\theta_{i} \right)=\text{Pr}\left( \text{lo}\text{g}_{10}\left( 500 \right)<\theta_{i}\leq\text{lo}\text{g}_{10}\left( 60,000 \right) \right) ,$$

$$\text{Pr}\left( \mathcal{G}_{\text{sub},i}=\text{NC} \mid\theta_{i} \right)=\text{Pr}\left( \mathcal{G}_{\text{bin},i}=\text{NC} \mid\theta_{i} \right)\text{=Pr}\left( \text{lo}\text{g}_{10}\left( 60,000 \right)<\theta_{i} \right) .$$

The translation of the continuous distribution of the ED_01_ into a discrete distribution is illustrated in Figure 1. The prior distribution for $\theta_{i}$ is chosen such that the prior probability of each GHS subcategory is one third. This necessarily induces a prior probability of two thirds for binary class 1 and 1/3 for binary class NC. Therefore, within the prior the SARA-ICE DA is biased towards predicting a chemical to be a sensitiser. This results in a small degree of *a priori* conservatism to estimates.


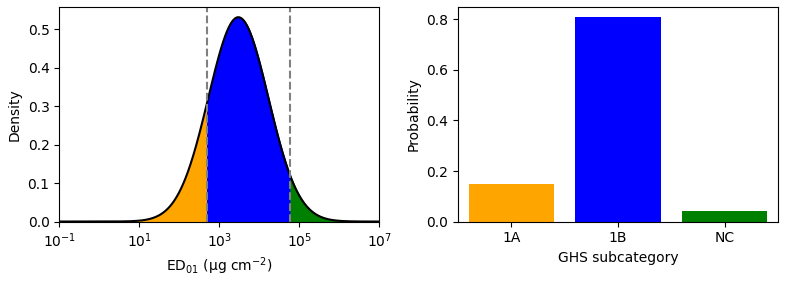


Figure 1 Example of translation of the distribution of the ED_01_ (left) to a discrete distribution for GHS subcategories. The areas under the density in the left plot are equal to the probability that the ED_01_ lies between/beyond the chosen classification thresholds of 500 µg cm^-2^ and 60,000 µg cm^-2^.

### GHS classifications

The previous section defines how GHS classification probabilities are calculated. This section defines a decision model to translate probabilities into actual classifications (or calls).

A probability threshold of 0.8 is proposed to enable a binary call (1/NC). The decision model for a binary call is as follows:

If $\text{Pr}\left( \mathcal{G}_{\text{bin},i}=1 \mid\theta_{i} \right)>0.8$, set $\mathcal{G}_{\text{bin},i}^{\text{call}}=1,$

else if $\text{Pr}\left( \mathcal{G}_{\text{bin},i}=NC \mid\theta_{i} \right)>0.8$, set $\mathcal{G}_{\text{bin},i}^{\text{call}}=NC,$

otherwise, $\mathcal{G}_{\text{bin},i}^{\text{call}}=\text{Inconclusive} .$

A second, less stringent threshold of 0.55 is used to enable a subcategory classification (1A/1B). If the binary call (1) is conclusive, then a subcategory call is made according to the following decision model:

If $\mathcal{G}_{\text{bin},i}^{\text{call}}=1$, calculate

$$\text{Pr}\left( \mathcal{G}_{\text{sub},i}=1A \mid\theta_{i},\mathcal{G}_{\text{bin},i}^{\text{call}}=1 \right)=\frac{\text{Pr}\left( \mathcal{G}_{\text{sub},i}=\text{1A} \mid\theta_{i} \right)}{\text{Pr}\left( \mathcal{G}_{\text{bin},i}=1 \mid\theta_{i} \right)} ,$$

$$\text{Pr}\left( \mathcal{G}_{\text{sub},i}=1B \mid\theta_{i},\mathcal{G}_{\text{bin},i}^{\text{call}}=1 \right)=\frac{\text{Pr}\left( \mathcal{G}_{\text{sub},i}=\text{1B} \mid\theta_{i} \right)}{\text{Pr}\left( \mathcal{G}_{\text{bin},i}=1 \mid\theta_{i} \right)} .$$

Then, if $\text{Pr}\left( \mathcal{G}_{\text{sub},i}=1A \mid\theta_{i},\mathcal{G}_{\text{bin},i}^{\text{call}}=1 \right)>0.55$, set $\mathcal{G}_{\text{sub},i}^{\text{call}}=1A$,

else if $\text{Pr}\left( \mathcal{G}_{\text{sub},i}=1B \mid\theta_{i},\mathcal{G}_{\text{bin},i}^{\text{call}}=1 \right)>0.55$, set $\mathcal{G}_{\text{sub},i}^{\text{call}}=1B$,

otherwise, $\mathcal{G}_{\text{sub},i}^{\text{call}}=\mathrm{Inconclusive}$.

else if $\mathcal{G}_{\text{bin},i}^{\text{call}}=NC,$ set $\mathcal{G}_{\text{sub},i}^{\text{call}}=NC$,

otherwise, $\mathcal{G}_{\text{sub},i}^{\text{call}}=\mathrm{Inconclusive}$.

Borderline classifications track the highest resolution classification possible – that is to say, if a subcategory classification can be made, then the borderline classification reflects that, if not, then the borderline classification reflects the binary classification. In the event that the binary call is inconclusive, we check whether subcategory 1A can be ruled out at a confidence level of 0.8. The decision model for the borderline classification is as follows:

If $\mathcal{G}_{\text{sub},i}^{\text{call}}\neq Inconclusive$, then $\mathcal{G}_{\text{border},i}^{\text{call}}=\mathcal{G}_{\text{sub},i}^{\text{call}}$,

else if $\mathcal{G}_{\text{bin},i}^{\text{call}}\neq\mathrm{Inconclusive}$, then $\mathcal{G}_{\text{border},i}^{\text{call}}=\mathcal{G}_{\text{bin},i}^{\text{call}}$,

else if $\text{Pr}\left( \mathcal{G}_{\text{sub},i}=\text{1A} \mid\theta_{i} \right)<0.2$, then $\mathcal{G}_{\text{border},i}^{\text{call}}=\mathrm{NC}/1B,$

otherwise, $\mathcal{G}_{\text{border},i}^{\text{call}}=\mathrm{Inconclusive}.$

# Basic model checks

## Influence of prior distributions

Unless explicitly explained, such as is the case for the ED_01_, prior distributions are chosen to be weakly regularising. This implies that we consider it undesirable if the prior distribution has a strong effect on posterior estimates. One approach to confirm this is to compare marginal posterior distributions against the prior for model parameters which we do not wish to impart too much influence. Figure 2 compares prior densities against histograms of such parameters. From this figure it can be seen that the posterior distribution of each parameter is not concentrating in the extreme tail of the prior, indicating that the chosen prior is not imparting any more than a soft regularisation on the posterior estimate. From this we conclude that estimates of model parameters would be unlikely to change in any appreciable manner if prior choices were relaxed further.


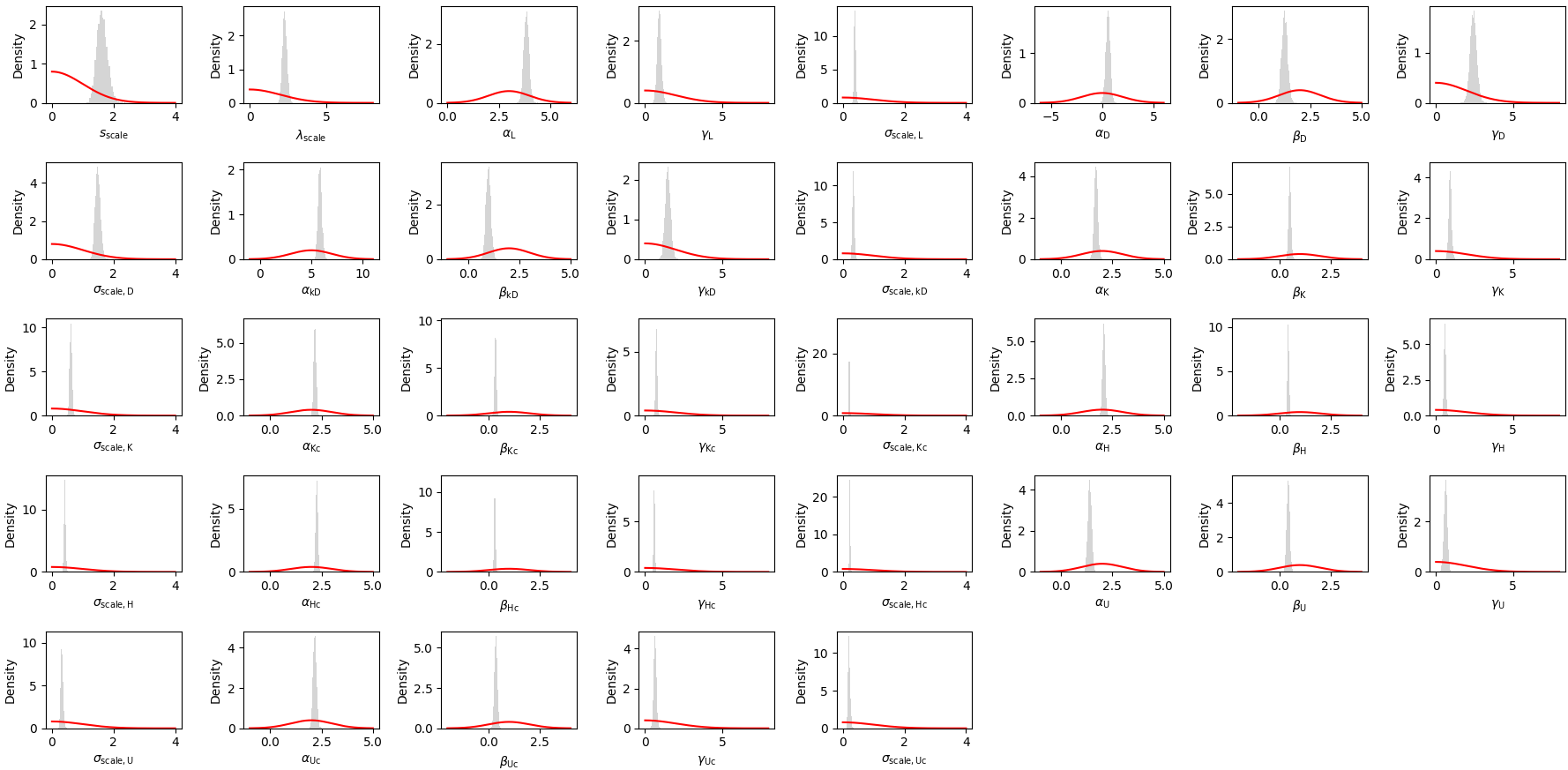


Figure 2 Comparison of prior densities (red) against marginal posterior distributions of chemical-agnostic parameters. For each parameter, observe that the posterior estimate is not concentrating in the extreme tail of the prior distribution.

## Correlations between latent mean estimates

The SARA-ICE model assumes linear relationships between latent parameters representing average inputs and the base-10 logarithm of the HPPT ED_01_ ($\theta_{i}$). In Figure 3, posterior expectations of $\theta_{i}\mid\mathcal{D}$ are plotted against posterior expectation of $\bar{y}_{i,s}\mid\mathcal{D}$, $s\in S$ for chemicals with at least one data point available for the corresponding input. The strongest correlation is obtained with the LLNA input, which is expected since the model parameter $\beta_{\text{L}}$ is fixed at a value of 1. The NAM input with the strongest association with human potency is reactivity rate measure from the kinetic DPRA. For the cell based NAMs, a stronger correlation is obtained with the assay-specific output (e.g., EC_1.5_ for the KeratinoSens^TM^ assay) than is obtained with the cytotoxicity measure.

Figure 4 displays the correlations between all non-HPPT inputs. The highest correlation is found between the h-CLAT potency input (minimum of CD86 EC_150_ and CD54 EC_200_, $\bar{y}_{i,H}\mid\mathcal{D}$) and the h-CLAT cytotoxicity input ($\bar{y}_{i,Hc}\mid\mathcal{D}$). Other pairs of inputs with notably strong correlations include DPRA depletion with kDPRA reactivity, cytotoxicity inputs from the KeratinoSens^TM^ and h-CLAT and U-Sens^TM^, U-Sens^TM^ potency and cytotoxicity, finally, and h-CLAT and U-Sens^TM^ potency. This last comparison is encouraging since these assays are considered to address the same key event (OECD TG 442E). Similarly, it is encouraging that the DPRA and kDPRA outputs are strongly associated.


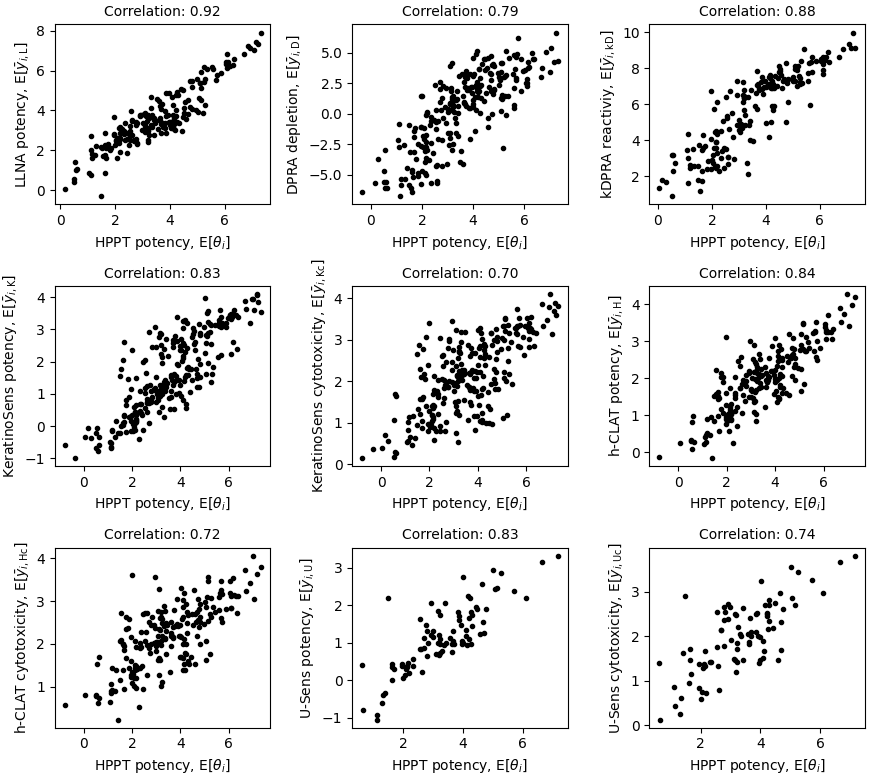


Figure 3 Correlation between posterior expectations of $\theta_{i}$ and $\bar{y}_{i,s}$ for $s\in S.$ Each point corresponds to a single chemical. Only chemicals with at least one data point for the given study type are displayed on the plot.


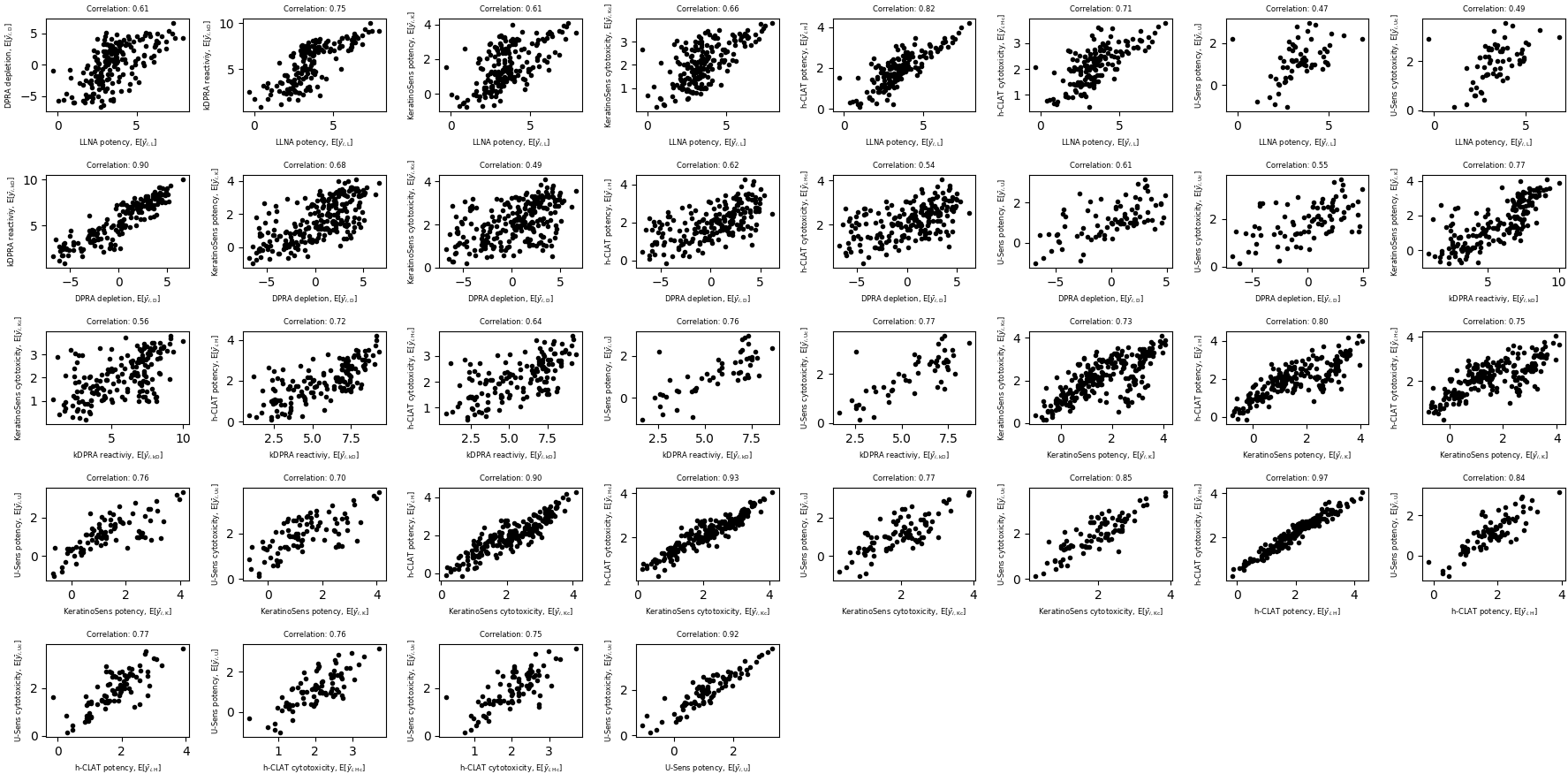


Figure 4 Correlation between each possible pair $\left( \text{E}\left[ \bar{y}_{i,s} \right],\text{E}\left[ \bar{y}_{i,t} \right] \right)$ for $s,t\in S$.

## Correlations between latent mean estimates and SARA-ICE inputs

Posterior expectations of the latent mean parameters $\bar{y}_{i,s}$ and individual inputs $y_{i,j,s}$ for $s\in S$ are compared in Figure 5. High correlations are obtained for all inputs indicating that mean estimates are largely consistent with the input data from which they are derived. The heterogenous structure of the SARA-ICE database implies some chemicals have multiple inputs available per input type. The variance of the scatter plots in the direction of the ordinate reflects the variability of the input type. This variance is noticeably smaller for cytotoxicity inputs in comparison to the specific inputs of the cell based NAMs. The kinetic DPRA also exhibits relatively low input variance, however this may be an artefact of limited repeat studies.

The informativeness of a single input for the purposes of estimating the ED_01_ is a function of both; how strong the relationship is in Figure 3, in addition to the strength of association in Figure 5. To assess the net effect of these sources of variability, posterior expectations of $\theta_{i}$ are compared against inputs $y_{i,j,s}$ for $s\in S$ in Figure 6. It is immediately noticeable that overall correlations between $\text{E}\left[ \theta_{i}\mid\mathcal{D} \right]$ and individual inputs are weaker than the interjoining correlations with latent mean estimates $\text{E}\left[ \bar{y}_{i,s}\mid\mathcal{D} \right]$. It is also apparent that correlations with cytotoxicity inputs are noticeably smaller, indicating that these are the least informative inputs.


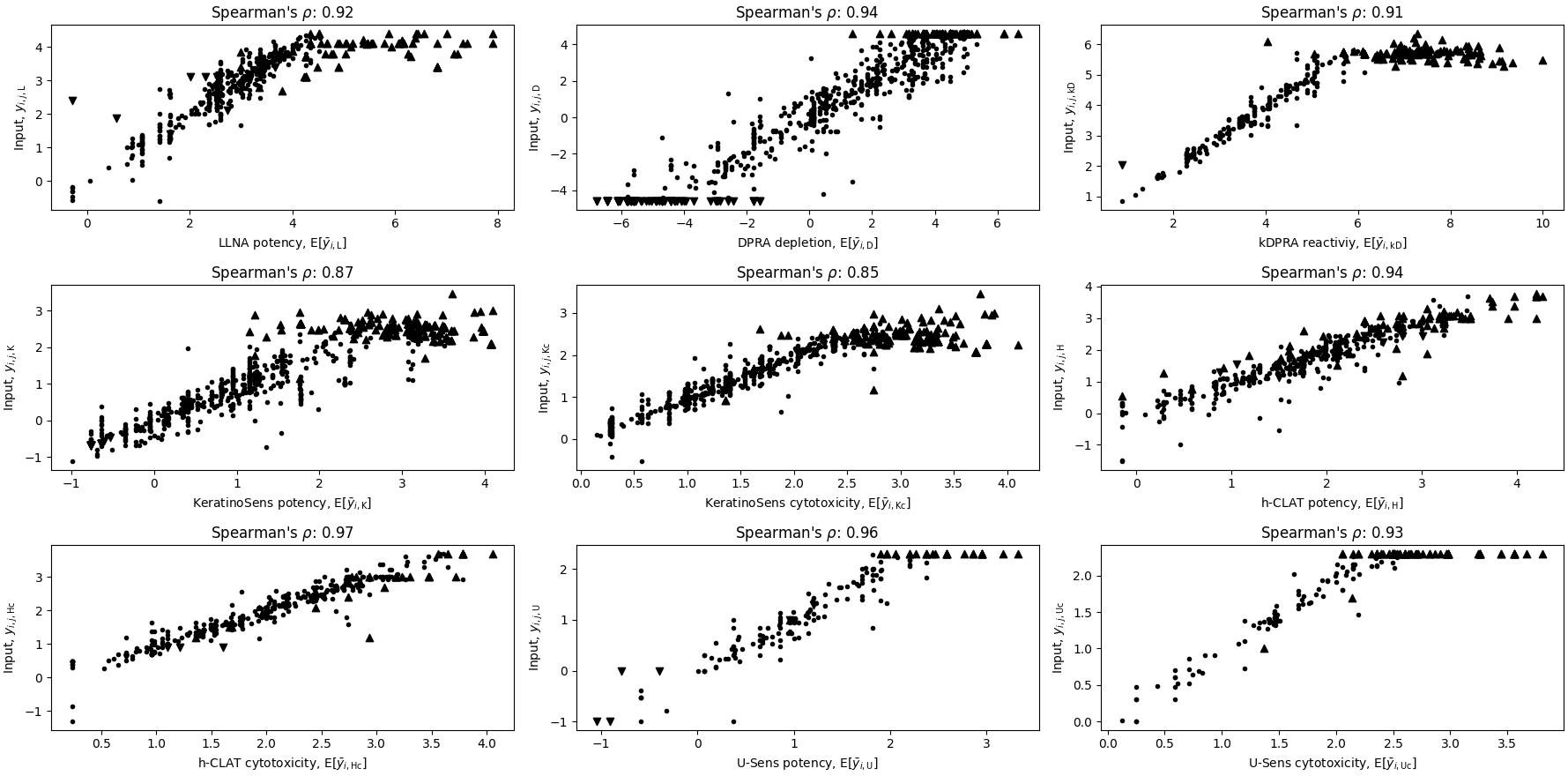


Figure 5 Correlations between posterior expectation of latent mean predictors $\text{E}\left[ \bar{y}_{i,s} \right]$ and transformed inputs $y_{i,j,s}$ for $s\in S$. Black points indicate regular inputs, upward pointing triangles indicate right-censored inputs and downward facing triangles indicate left-censored inputs. Correlation is measured using Spearman’s ρ due to the “kinks” induced by the typical censoring points.

*
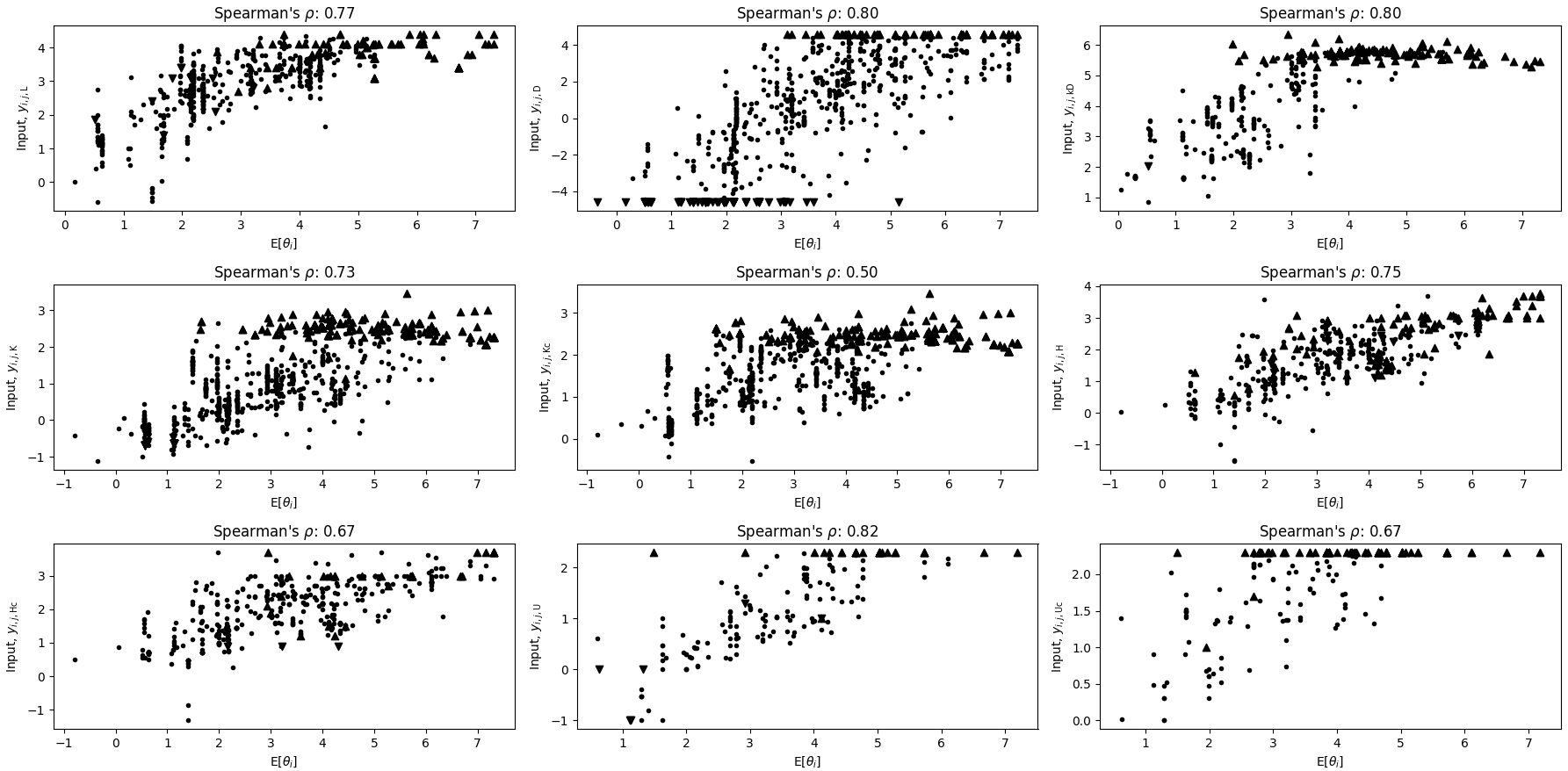
*

Figure 6 Correlation between posterior expectations of $\theta_{i}$ and inputs $y_{i,j,s}$ for $s\in S$. Black points indicate regular inputs, upward pointing triangles indicate right-censored inputs and downward facing triangles indicate left-censored inputs. Correlation is measured using Spearman’s ρ due to the “kinks” induced by the typical censoring points.

## Accuracy of the SARA-ICE production model

The accuracy of the SARA-ICE production model was checked by comparing ED_01_ estimates generated using the full model against those generated using the production model. The SARA-ICE model was fit to the full database and the 5^th^, 50^th^ and 95^th^ percentiles of the marginal posterior distribution of each $\theta_{i}$, $i=1,\ldots,434$ were computed using a sample of 10,000 posterior draws. For each chemical in the database, we extracted the chemical-specific information and estimated $\hat{\theta_{i}}$ from the SARA-ICE production model. The 5^th^, 50^th^ and 95^th^ percentiles of each $\hat{\theta_{i}}$ were computed. Percentile estimates from both versions of the model are compared in Figure 7. Correlation between percentile estimates is very close to 1 indicating both models for all practical purposes result in the same estimates.


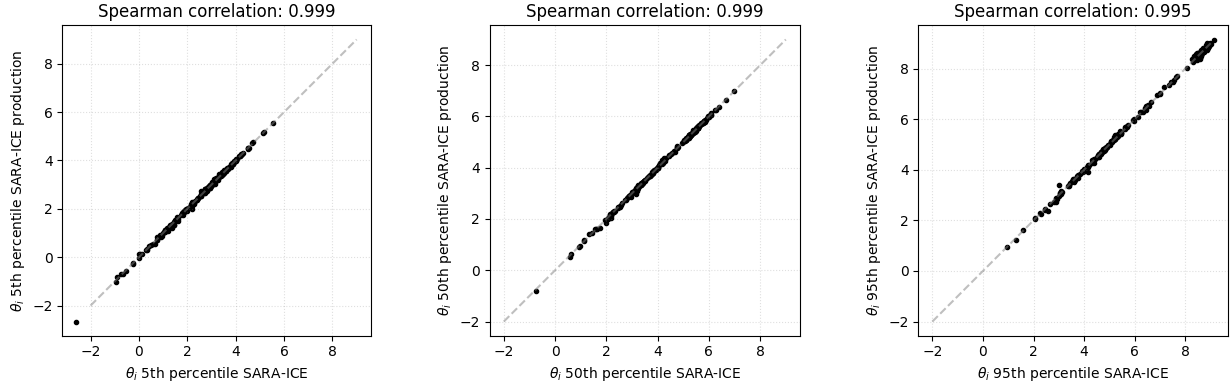


Figure 7 Comparisons of percentiles (5th, 50th and 95th) of $\theta_{i}$ when estimated using SARA-ICE versus SARA-ICE production.

# References

Carpenter, B., Gelman, A., Hoffman, M. D., Lee, D., Goodrich, B., Betancourt, M., . . . Riddell, A. (2017). Stan: a probabilistic programming language. *Grantee Submission, 76*(1), 1-32.

Hoffmann, S., Alépée, N., Gilmour, N., Kern, P. S., van Vliet, E., Boislève, F., . . . Kühnl, J. (2022). Expansion of the Cosmetics Europe skin sensitisation database with new substances and PPRA data. *Regulatory Toxicology and Pharmacology, 131*, 105169.

Kligman, A. M. (1966). The identification of contact allergens by human assay: III. The maximization test: A procedure for screening and rating contact sensitizers. *Journal of Investigative Dermatology, 47*(5), 393-409.

Kligman, A. M., & Epstein, W. (1975). Updating the maximization test for identifying contact allergens. *Contact Dermatitis, 1*(4), 231-239.

Lewandowski, D., Kurowicka, D., & Joe, H. (2009). Generating random correlation matrices based on vines and extended onion method. *Journal of Multivariate Analysis, 100*(9), 1989-2001. doi:<https://doi.org/10.1016/j.jmva.2009.04.008>

OECD. (2010). *Test No. 429: Skin Sensitisation*.

OECD. (2018a). *Test No. 442D: In Vitro Skin Sensitisation*.

OECD. (2018b). *Test No. 442E: In Vitro Skin Sensitisation*.

OECD. (2020). *Test No. 442C: In Chemico Skin Sensitisation*.

OECD. (2021a). *ANNEX 4: REPORT OF THE HUMAN DATA SUB-GROUP ON THE CURATION AND*

*EVALUATION OF THE HUMAN REFERENCE DATA AND THE DERIVATION OF*

*ASSOCIATED SUBSTANCE CLASSIFICATIONS*.

OECD. (2021b). Supporting Document to the OECD Guideline 497 on Defined Approaches for Skin Sensitisation. Retrieved from <https://www.oecd.org/officialdocuments/publicdisplaydocumentpdf/?cote=ENV/CBC/MONO(2021)11&docLanguage=En>

Politano, V. T., & Api, A. M. (2008). The Research Institute for Fragrance Materials' human repeated insult patch test protocol. *Regul Toxicol Pharmacol, 52*(1), 35-38. doi:10.1016/j.yrtph.2007.11.004

Reynolds, J., Gilmour, N., Baltazar, M. T., Reynolds, G., Windebank, S., & Maxwell, G. (2022). Decision making in next generation risk assessment for skin allergy: Using historical clinical experience to benchmark risk. *Regulatory Toxicology and Pharmacology, 134*, 105219. doi:<https://doi.org/10.1016/j.yrtph.2022.105219>

Reynolds, J., MacKay, C., Gilmour, N., Miguel-Vilumbrales, D., & Maxwell, G. (2019). Probabilistic prediction of human skin sensitiser potency for use in next generation risk assessment. *Computational Toxicology, 9*, 36-49.

Ryan, C. A., Chaney, J. G., Frank Gerberick, G., Kern, P. S., Dearman, R. J., Kimber, I., & Basketter, D. A. (2007). Extrapolating local lymph node assay EC3 values to estimate relative sensitizing potency. *Cutaneous and ocular toxicology, 26*(2), 135-145.

UN. (2021). *Globally Harmonised System of Classification and Labelling of Chemicals (GHS)*.
