# Supplementary material for: The skin allergy risk assessment-integrated chemical environment (SARA-ICE) defined approach to derive points of departure for skin sensitization
Source: Curr Res Toxicol. 2024 Dec 14;8:100205. doi: 10.1016/j.crtox.2024.100205 (PMC11719337; doi:10.1016/j.crtox.2024.100205)
Supplement: Supplementary Data 2 [file mmc2.docx]

Performance of SARA-ICE against OECD benchmark GHS – Cross validation

# Acronyms

SARA – skin allergy risk assessment

ICE – integrated chemical environment

DA – defined approach

HPPT – human predictive patch test

HRIPT – human repeat insult patch test

HMT – human maximisation test

LLNA – local lymph node assay

DPRA – direct peptide reactivity assay

kDPRA – kinetic direct peptide reactivity assay

GHS – UN Globally Harmonized System of Classification and Labelling of Chemicals

OECD – Organisation for Economic Co-operation and Development

NAM – new approach method

# Scope

Assess the performance of SARA-ICE for GHS classification using the OECD benchmark dataset as a reference. The reference dataset is provided as annex 2 of (OECD, 2021).

# Overlap of OECD benchmark dataset with SARA-ICE database

There are 196 chemicals within the OECD benchmark dataset and 187 of these are present within the SARA-ICE database with varying amounts of associated data. For example, cinnamic alcohol has 75 chemical-specific studies (25 HPPT, 3 LLNA, 8 DPRA, 1 kDPRA, 29 KeratinoSens^TM^, 6 h-CLAT and 3 U-Sens^TM^) whilst 2-Methyldecanenitrile has only two studies (1 LLNA and 1 kDPRA).

# Predicting GHS classification with SARA-ICE

Any combination of chemical-specific inputs may be used with the SARA-ICE model to generate a probabilistic prediction of GHS classification. Furthermore, due to the overlap between chemical sets, it is desirable to use leave-one-chemical-out strategies when generating chemical-specific predictions. Performance statistics are calculated following each of the scenarios listed below:

1. **SARA-ICE with full database**. The SARA-ICE model is trained on the SARA-ICE database and GHS classification probabilities are extracted for OECD benchmark chemicals. The number of studies underpinning each prediction is heterogenous and covers all possible inputs. For OECD benchmark chemicals which are not present in the SARA-ICE database, these chemicals will be included when training the model but, because they have no chemical-specific inputs available, GHS classifications should be inconclusive for this set.
2. **SARA-ICE production with OCED NAM inputs only**. To create a version of SARA-ICE that can be used in an on-demand service (such as a web interface), we first train the model on the full database and then create an analytical approximation to the marginal posterior distribution of all chemical-agnostic parameters (details can be found in Supplementary Information (1)). This distribution is then used as the prior within SARA-ICE, which can be thought of as a production version of SARA-ICE. It takes a matter of seconds to generate a prediction for a single chemical using this version of the model (compared to around an hour when training on the full database). The SARA-ICE production model is used to generate GHS classifications for each OECD benchmark chemical using only the NAM data provided in the OECD benchmark database plus kDPRA studies available in (Natsch & Gerberick, 2022). This reference results in 171 out of 196 benchmark chemicals having a single kDPRA study available.
3. **10-fold cross validation to assess stability of predictions with OECD NAM inputs**. A tenth of the OECD benchmark chemicals are randomly chosen and their data in the SARA-ICE database is swapped for only the studies provided in the OECD benchmark dataset plus a kDPRA from (Natsch & Gerberick, 2022), if available. The SARA-ICE model is trained on the modified database and GHS classifications for the chemicals with modified data are extracted. This procedure is repeated 9 times such that a single GHS classification is generated for all 196 chemicals. Since the choice of chemicals to be left out at each fold is random, the entire procedure is repeated 20 times to assess stability of predictions.

# Results

## All available inputs (full database) with SARA-ICE

### Chemicals not in the SARA-ICE database

There are 9 OECD benchmark chemicals which are not included in the SARA-ICE database. In the absence of chemical-specific information, GHS classification probabilities for these chemicals are approximately equal to the prior distribution probabilities of P(1A)=P(1B)=P(NC)=1/3. These probabilities do not reach the confidence levels for classification, therefore are all GHS classifications are **inconclusive** for this set.

*Recap: probability thresholds for a conclusive classification are currently set at 0.8 for the binary classification and 0.55 for the subcategory classification of 1A/1B when a binary call of sensitiser has been made. These thresholds are imposed for all classifications, irrespective of the data type(s) used to generate the classification probabilities.*

### Human and LLNA concordant subclassifications

#### Subcategory NC

There are 2 chemicals in the OECD benchmark dataset for which human and LLNA subclassifications agree on NC. These are also classified as NC by SARA-ICE.

#### Subcategory 1A

There are 9 chemicals in the OECD benchmark dataset for which human and LLNA subclassifications agree on 1A. These are also classified as 1A by SARA-ICE.

#### Subcategory 1B

There are 17 chemicals in the OECD benchmark dataset for which human and LLNA subclassifications agree on 1B. 13 of these are also classified as 1B by SARA-ICE. Of the remaining four, 2 have inconclusive subcategory classifications (pyridine and ethyl acrylate) and 2 have classifications of 1A (penicillin G and thiram). Pyridine does not have *in vivo* data in the SARA-ICE database and it’s NAM data is mixed between negative DPRA and kDPRA and positive KeratinoSens^TM^ and h-CLAT results. The binary classification is inconclusive for this chemical which automatically induces an inconclusive subcategory classification. Ethyl acrylate has at least one study for every input and has a near-certain binary classification of sensitiser. With a median ED_01_ of 460 µg cm^-2^ its subcategory probabilities are close to 0.5 for 1A and 1B (for which the boundary is 500 µg cm^-2^). For penicillin G and thiram the SARA-ICE subcategory classification is 1A. The human data for penicillin G includes HPPT studies with sensitisation reported at doses as low as 60 µg cm^-2^ and for thiram the NAM data includes near full depletion in the DPRA, and responses observed at the lowest dose tested in the cell-based assays.

### Human and LLNA discordant subclassifications

#### Human subcategory NC, LLNA subcategory 1A/1B/NA

There are 9 chemicals with a human subcategory of NC and a non-matching LLNA subcategory (6 1B and 3 unclassified). The SARA-ICE GHS classification is inconclusive for 7 of these. The factors preventing a conclusive prediction primarily stem from mixed results in the data used to generate the classification probabilities. For example, for DMSO, the probability of NC is 0.73 which is slightly lower than the threshold of 0.8 required to call NC. DMSO has a positive LLNA which reduces confidence in the NC classification within the weight-of-evidence. Hydrocortisone has a single HMT with no one of 25 individuals sensitised and a negative h-CLAT study but exhibits high depletion with the lysine peptide in the DPRA and is positive in KeratinoSens^TM^ assay. With a probability of 0.66, NC is the most likely GHS classification, but the confidence is not high enough to make a conclusive call.

A subcategory classification of 1B was made for sodium lauryl sulfate owing largely to several positive LLNA and NAM results. A subcategory classification of NC was made for 4-aminobenzoic acid, which has human classification NC and unassigned LLNA classification.

#### Human subcategory 1A, LLNA subcategory 1B/NC/NA

There are 12 chemicals with a human subcategory of 1A and a non-matching LLNA subcategory (7 1B and 5 unclassified). The SARA-ICE subcategory classification is 1A for 9 of these. The subclassification is 1B (matching the LLNA) for butyl glycidyl ether. A subcategory classification of 1B is made for 6-methylhepta-3,5-dien-2-one (LLNA classification is borderline NC/1B with subcategory unclassified). There are no human studies in the SARA-ICE database for this chemical. Both binary and subcategory classifications for benzaldehyde are inconclusive, however, borderline subcategory matches the LLNA borderline classification of NC/1B (LLNA subcategory undefined).

#### Human subcategory 1B, LLNA subcategory 1A/NC/Unclassified

There are 14 chemicals with human subcategory of 1B and a non-matching LLNA subcategory (3 1A, 3 NC and 8 unclassified). 6 of these have SARA-ICE subcategory 1B (LLNA unclassified in each). 3 chemicals have SARA-ICE subcategory 1A (also matching the LLNA subcategory in each case). Benzyl alcohol has SARA-ICE subcategory NC, matching the LLNA subcategory of NC. This is also in line with a recent publication recommending that benzyl alcohol should not be classified as 1B (Geier et al., 2022). All HPPT studies in the SARA-ICE database report no individuals sensitised for this chemical and the single LLNA study is negative. Finally, four chemicals have SARA-ICE inconclusive classifications, one of which is a chemical without any chemical-specific data. Two of the SARA-ICE unclassified are also unclassified in the LLNA.

### Remaining human binary classifications

There are three additional chemicals with a human binary classification (all class 1) but with no subcategory classification. Both the LLNA binary classification and SARA-ICE binary classification agree. Two LLNA subcategory classifications are assigned, both 1B. The SARA-ICE subcategory agrees with one (3,4-dihydrocoumarin) and disagrees with the second (citral). For citral, the subcategory classification of 1A is due to the presence of two HPPT studies with sensitisation observed at 65 µg cm^-2^.

### LLNA subcategory classifications (unassigned human classifications)

#### LLNA subcategory classification NC

There are 28 chemicals with LLNA subcategory classification NC and no human classification. SARA-ICE classifications are inconclusive for 13 of these. Of the remaining 15 chemicals, the SARA-ICE subcategory classification is NC for 14. Methyl 3-bromoproprionate has a subcategory classification of 1B from SARA-ICE – whilst the LLNA is negative up to concentrations of 50%, the single DPRA is reported to have 97% depletion on the cysteine peptide, the single kDPRA has a relatively fast depletion rate (log Kmax = -1.64) and the single KeratinoSens^TM^ study is positive (EC_1.5_ = 41 µM).

#### LLNA subcategory classification 1A

There are 26 chemicals with an LLNA subcategory of 1A and no human classification. The SARA-ICE subcategory classification is 1A for 24 of these. The SARA-ICE subcategory classification is 1B for 1-naphthol and cyclohexane-1,2-dicarboxylic anhydride, owing to relatively low depletion in the DPRA and some negative cell-based NAMs.

#### LLNA subcategory classification 1B

There are 53 chemicals with LLNA subcategory classification 1B and no human classification. The SARA-ICE subclassification is inconclusive for 18 of these and in agreement with 1B for 30. The SARA-ICE subcategory classification is 1A for four chemicals and NC for alpha-tocopherol. Alpha-tocopherol does not have *in vivo* data in the SARA-ICE database and all but one NAM inputs are negative (the KeratinoSens^TM^ is positive).

### Remaining LLNA binary classifications

There are 5 chemicals with an LLNA binary classification, but no LLNA subcategory classification or human classification. The binary classification is 1 in each case and the SARA-ICE binary classification is 1 for all chemicals.

### Remaining chemicals

The final set of 11 chemicals includes those with a borderline classification only. For each chemical, the LLNA borderline classification is NC/1B and where available, the human borderline classification agrees. The SARA-ICE binary and subcategory classifications are inconclusive for 7 of these. However, the probability of a borderline classification of NC/1B is approximately 0.9 or greater for all 7 chemicals with an inconclusive binary prediction. SARA-ICE subcategory classifications are NC for methyl dihydrojasmonate, sulfanilic acid and ethylene brassylate. Propyl paraben has a SARA-ICE subcategory classification of 1B.

### Summary

#### Inconclusive rate

187 / 196 chemicals have more than one chemical-specific input available in the SARA-ICE dataset. Binary predictions were conclusive for 142 of these, whilst predictions for the remaining 45 chemicals were inconclusive. The binary inconclusive rate from the heterogenous SARA-ICE database inputs is 24%. There are 50 inconclusive subcategory predictions giving the marginally higher subcategory inconclusive rate of 27%.

#### Classification accuracy

The following table summarises the binary classification metrics against human and LLNA benchmarks.

Table 1 Classification concordance of SARA-ICE, using all available inputs, against reference classifications.

| **Human, Θ_bin_ = 0.80** | **SARA-ICE, GHS 1** | **SARA-ICE, GHS NC** | **Inconclusive** | **Total** |
| --- | --- | --- | --- | --- |
| **Reference, GHS 1** | 48 | 1 | 6 | 55 |
| **Reference, GHS NC** | 1 | 3 | 7 | 11 |
| **Total** | 49 | 4 | 13 | 66 |
| Sensitivity: 98%  Specificity: 75%  **Balanced accuracy: 86%**  OECD Reference, GHS 1 inconclusive: 11%  OECD Reference, GHS NC inconclusive: 64% | | | | |
| **LLNA, Θ_bin_ = 0.80** | **SARA-ICE, GHS 1** | **SARA-ICS, GHS NC** | **Inconclusive** | **Total** |
| **Reference, GHS 1** | 113 | 1 | 21 | 135 |
| **Reference, GHS NC** | 1 | 17 | 15 | 33 |
| **Total** | 114 | 18 | 36 | 168 |
| Sensitivity: 99%  Specificity: 94%  **Balanced accuracy: 97%**  OECD Reference, GHS 1 inconclusive: 16%  OECD Reference, GHS NC inconclusive: 45% | | | | |

This table summarises the subcategory classification metrics.

| **Human, Θ_bin_ = 0.80, Θ_sub_=0.55** | **SARA-ICE, GHS 1A** | **SARA-ICE, GHS 1B** | **SARA-ICE, GHS NC** | **Inconclusive** | **Total** |
| --- | --- | --- | --- | --- | --- |
| **OECD** Reference, GHS **1A** | 18 | 2 | 0 | 1 | 21 |
| **OECD** Reference, GHS **1B** | 5 | 19 | 1 | 6 | 31 |
| **OECD** Reference, GHS **NC** | 0 | 1 | 3 | 7 | 11 |
| **Total** | 23 | 22 | 4 | 14 | 63 |
| Sensitivity 1A: 90%, Specificity 1A: 83%, Balanced accuracy 1A: 86%  Sensitivity 1B: 76%, Specificity 1B: 88%, Balanced accuracy 1B: 82%  Sensitivity NC: 75%, Specificity NC: 98%, Balanced accuracy NC: 86%  **Average balanced accuracy: 85%**  OECD Reference, GHS 1A inconclusive: 5%  OECD Reference, GHS 1B inconclusive: 19%  OECD Reference, GHS NC inconclusive: 64% | | | | | |
| **LLNA, Θ_bin_ = 0.80, Θ_sub_=0.55** | **SARA-ICE, GHS 1A** | **SARA-ICE, GHS 1B** | **SARA-ICE, GHS NC** | **Inconclusive** | **Total** |
| **OECD** Reference, GHS **1A** | 36 | 2 | 0 | 0 | 38 |
| **OECD** Reference, GHS **1B** | 13 | 46 | 1 | 25 | 85 |
| **OECD** Reference, GHS **NC** | 0 | 1 | 17 | 15 | 33 |
| **Total** | 49 | 49 | 18 | 40 | 156 |
| Sensitivity 1A: 95%, Specificity 1A: 83%, Balanced accuracy 1A: 89%  Sensitivity 1B: 77%, Specificity 1B: 95%, Balanced accuracy 1B: 86%  Sensitivity NC: 94%, Specificity NC: 99%, Balanced accuracy NC: 97%  **Average balanced accuracy: 90%**  OECD Reference, GHS 1A inconclusive: 0%  OECD Reference, GHS 1B inconclusive: 29%  OECD Reference, GHS NC inconclusive: 45% | | | | | |

## OECD NAM inputs with SARA-ICE production

We next consider generating GHS classifications using OECD reported NAM inputs in addition to kDPRA data for 171 of the 196 OECD benchmark chemicals. Throughout this section we’ll compare predictions against the previous set of SARA-ICE predictions made using the full database and the OECD benchmarks.

### Inconclusive predictions

For several chemicals, the new GHS prediction is made without the previously available *in vivo* data in SARA-ICE. This results in an, on average, increase in the inconclusive rate when generating predictions using NAM data only. There are 34 chemicals for which a binary GHS call was made using all available data in the SARA-ICE database but for which the call becomes inconclusive when using NAM data only. There are a further 11 chemicals which have a conclusive subcategory classification using the data in SARA-ICE but inconclusive subcategory classification using NAM inputs only.

Conversely, there are 14 chemicals which were previously inconclusive binary but are conclusive using only NAM inputs. Of these, sulfanilamide, DMSO and kanamycin have defined human GHS classifications and discordant LLNA classifications. The NAM-only GHS prediction is NC for all three, which agrees with the LLNA benchmark for sulfanilamide and kanamycin and agrees with the human benchmark for DMSO. LLNA benchmarks are defined for 9 of the remaining 11 chemicals. The SARA-ICE NAM binary classifications agree with 5 of these, whilst subcategory classifications agree for 4. Binary classifications disagree for 1-Iodohexane (1B using NAM inputs), methyl pyruvate and anisyl alcohol (both NC using NAM inputs).

There are an additional 5 chemicals which previously had a conclusive binary prediction but inconclusive subcategory prediction. For 3-aminophenol the NAM-only binary prediction is inconclusive. NAM predictions for methyl methanesulphonate are also conclusive binary and inconclusive subcategory. A conclusive subcategory prediction was made using NAM data only for abietic acid, methyl acrylate and ethyl acrylate. In each case this was 1A which disagrees with all benchmarks of 1B.

### Conclusive subcategory predictions

There are 93 chemicals with a conclusive subcategory classification using both sets of SARA-ICE inputs.

#### Subcategory 1A

Within this set of 93 chemicals, 48 have a subcategory prediction of 1A given inputs in the SARA-ICE database and the prediction using NAM only inputs agrees for 43 chemicals. 2-Nitro-p-phenylenediamine and DNBS, sodium salt have SARA-ICE subcategory classifications of 1B using NAM inputs, which disagree with the 1A LLNA benchmarks. 5-Amino-o-cresol and dibenzoyl peroxide also have 1B classifications using NAM inputs, which agrees with LLNA benchmark of 1B for 5-amino-o-cresol (no human benchmark defined) and agrees with the human benchmark of 1B for dibenzoyl peroxide (LLNA benchmark is 1A). Penicillin G has a NAM classification of NC, which contradicts the classification of 1A obtained with the *in vivo* data in the SARA-ICE database and the benchmarks of 1B.

#### Subcategory 1B

There are 29 chemicals within the set of 93 that have a subcategory prediction of 1B given inputs in the SARA-ICE database. The SARA-ICE classification using NAMs results in a concordant prediction for 24 chemicals. Imidazolidinyl urea, farnesal and 2-ethylhexyl acrylate have a NAM subcategory classification of 1A whilst allyl phenoxyacetate and squaric acid have a subcategory classification of NC. The benchmark, where available, is 1B for all 5 of these chemicals.

#### Subcategory NC

The remaining 16 chemicals of the 93 have SARA-ICE classifications of NC, with no disagreements between input sets. All benchmark classifications are NC except for benzyl alcohol which has a classification of 1B (discussed above).

### Summary

#### SARA-ICE classification concordance

We first report concordance of SARA-ICE classifications when generated using a) data in the SARA-ICE database and b) NAM data only obtained from the OECD benchmark dataset. For the subset of chemicals for which conclusive classifications were made using both sets of inputs, the concordance between classifications is high.

Table 2 Summary of classification concordance between SARA-ICE using all available inputs versus SARA-ICE using OECD NAM inputs only.

| **SARA-ICE database vs SARA-ICE NAM, Θ_bin_ = 0.80** | **SARA-ICE NAM, GHS 1** | **SARA-ICE NAM, GHS NC** | **SARA-ICE NAM, Inconclusive** | **Total** |
| --- | --- | --- | --- | --- |
| **SARA-ICE database, GHS 1** | 89 | 3 | 28 | 120 |
| **SARA-ICE database, GHS NC** | 0 | 16 | 6 | 22 |
| **SARA-ICE database, Inconclusive** | 4 | 10 | 40 | 54 |
| **Total** | 93 | 29 | 74 | 196 |
| Sensitivity: 97%  Specificity: 100%  **Balanced accuracy: 98%**  Total inconclusive SARA-ICE database: 28%  Total inconclusive SARA-ICE NAM: 38% | | | | |

| **SARA-ICE database vs SARA-ICE NAM, Θ_bin_ = 0.80, Θ_sub_=0.55** | **SARA-ICE NAM, GHS 1A** | **SARA-ICE NAM, GHS 1B** | **SARA-ICE NAM, GHS NC** | **SARA-ICE NAM, Inconclusive** | **Total** |
| --- | --- | --- | --- | --- | --- |
| **SARA-ICE database, GHS 1A** | 43 | 4 | 1 | 8 | 56 |
| **SARA-ICE database, GHS 1B** | 3 | 24 | 2 | 30 | 59 |
| **SARA-ICE database, GHS NC** | 0 | 0 | 16 | 6 | 22 |
| **SARA-ICE database, Inconclusive** | 5 | 2 | 10 | 42 | 59 |
| **Total** | 51 | 30 | 29 | 86 | 196 |
| Sensitivity 1A: 90%, Specificity 1A: 93%, Balanced accuracy 1A: 91%  Sensitivity 1B: 83%, Specificity 1B: 92%, Balanced accuracy 1B: 88%  Sensitivity NC: 100%, Specificity NC: 96%, Balanced accuracy NC: 98%  **Average balanced accuracy: 92%**  Total inconclusive SARA-ICE database: 30%  Total inconclusive SARA-ICE NAM: 44% | | | | | |

#### Benchmark classification concordance

Classification metrics for SARA-ICE NAM-based predictions against OECD benchmarks are presented in the tables below. Overall, the inconclusive rate is higher when using NAM inputs only. Binary classification performance against the human benchmarks is improved, however, this improvement is due to changes in just two predictions. The binary prediction of 1 for sodium lauryl sulfate using all data in the SARA-ICE database becomes inconclusive when estimated using NAM data only whilst the inconclusive classification for DMSO becomes NC with NAM data only. This increases the specificity from 75% to 100%, resulting in a 9-percentage point increase in the balanced accuracy. On the other hand, balanced accuracy against the LLNA benchmarks decreases from 97% to 92%.

Average balanced accuracy against the human subcategory benchmarks decreases from 85% to 83% and against the LLNA benchmarks the decrease is from 90% to 82%.

Table 3 Binary classification performance for SARA-ICE production against human and LLNA benchmarks.

| **Human, Θ_bin_ = 0.80** | **SARA-ICE, GHS 1** | **SARA-ICE, GHS NC** | **SARA-ICE, Inconclusive** | **Total** |
| --- | --- | --- | --- | --- |
| **OECD** Reference, GHS **1** | 37 | 4 | 14 | 55 |
| **OECD** Reference, GHS **NC** | 0 | 4 | 7 | 11 |
| **Total** | 37 | 8 | 21 | 66 |
| Sensitivity: 90%  Specificity: 100%  **Balanced accuracy: 95%**  OECD Reference, GHS 1 inconclusive: 25%  OECD Reference, GHS NC inconclusive: 64% | | | | |
| **LLNA, Θ_bin_ = 0.80** | **SARA-ICE, GHS 1** | **SARA-ICE, GHS NC** | **SARA-ICE, Inconclusive** | **Total** |
| **OECD** Reference, GHS **1** | 87 | 6 | 42 | 135 |
| **OECD** Reference, GHS **NC** | 2 | 19 | 12 | 33 |
| **Total** | 89 | 25 | 54 | 168 |
| Sensitivity: 94%  Specificity: 90%  **Balanced accuracy: 92%**  OECD Reference, GHS 1 inconclusive: 31%  OECD Reference, GHS NC inconclusive: 36% | | | | |

Table 4 Subcategory classification performance for SARA-ICE production against human and LLNA benchmarks.

| **Human, Θ_bin_ = 0.80, Θ_sub_=0.55** | **SARA-ICE, GHS 1A** | **SARA-ICE, GHS 1B** | **SARA-ICE, GHS NC** | **SARA-ICE, Inconclusive** | **Total** |
| --- | --- | --- | --- | --- | --- |
| **OECD** Reference, GHS **1A** | 14 | 2 | 0 | 5 | 21 |
| **OECD** Reference, GHS **1B** | 4 | 9 | 4 | 14 | 31 |
| **OECD** Reference, GHS **NC** | 0 | 0 | 4 | 7 | 11 |
| **Total** | 18 | 11 | 8 | 26 | 63 |
| Sensitivity 1A: 88%, Specificity 1A: 81%, Balanced accuracy 1A: 84%  Sensitivity 1B: 53%, Specificity 1B: 90%, Balanced accuracy 1B: 71%  Sensitivity NC: 100%, Specificity NC: 88%, Balanced accuracy NC: 94%  **Average balanced accuracy: 83%**  OECD Reference, GHS 1A inconclusive: 24%  OECD Reference, GHS 1B inconclusive: 45%  OECD Reference, GHS NC inconclusive: 64% | | | | | |
| **LLNA, Θ_bin_ = 0.80, Θ_sub_=0.55** | **SARA-ICE, GHS 1A** | **SARA-ICE, GHS 1B** | **SARA-ICE, GHS NC** | **SARA-ICE, Inconclusive** | **Total** |
| **OECD** Reference, GHS **1A** | 28 | 4 | 0 | 6 | 38 |
| **OECD** Reference, GHS **1B** | 16 | 22 | 5 | 42 | 85 |
| **OECD** Reference, GHS **NC** | 0 | 1 | 19 | 13 | 33 |
| **Total** | 44 | 27 | 24 | 61 | 156 |
| Sensitivity 1A: 88%, Specificity 1A: 75%, Balanced accuracy 1A: 81%  Sensitivity 1B: 51%, Specificity 1B: 90%, Balanced accuracy 1B: 71%  Sensitivity NC: 95%, Specificity NC: 93%, Balanced accuracy NC: 94%  **Average balanced accuracy: 82%**  OECD Reference, GHS 1A inconclusive: 16%  OECD Reference, GHS 1B inconclusive: 49%  OECD Reference, GHS NC inconclusive: 39% | | | | | |

## Stability of classifications to changes to the SARA-ICE database with SARA-ICE production

The performance metrics in the previous section were generated by first training the SARA-ICE model on the full database, then using the parameter estimates to define the SARA-ICE production model, followed by generating predictions for each benchmark chemical using the NAM data provided in the OECD benchmark dataset.

The estimated classification performance is dependent on the data in the SARA-ICE database. If the database is changed slightly, model outputs will also change which in turn may modify the performance metrics. This section aims to quantify the magnitude of that change. That is, we aim to characterise the variability in the performance metrics following modifications to the SARA-ICE database.

A 10-fold cross-validation approach is used. The set of chemicals in the OECD benchmark dataset is randomly partitioned into 10 disjoint subsets whose union makes up the full set of 196 benchmarks. Using an iterative procedure, the chemicals within each subset are removed from the SARA-ICE database, and the model trained on the remaining database chemicals using all available data (*in vivo* and *in vitro*). Parameter estimates are then used to define a SARA-ICE production model. The SARA-ICE production model is used to generate ED_01_ estimates and GHS classification probabilities for the chemicals in the removed subset. The procedure is repeated for each random subset of the OECD benchmark list until GHS classification probabilities have been generated for all chemicals. As the partitioning is random, the entire procedure is repeated 20 times to build 20 different production models, each of which is used to generate a which builds up a distribution of predictions for each chemical. Summary performance metrics such as sensitivity, specificity and balanced accuracy are calculated once per repeat; the distribution of these is discussed below.

### Variability in GHS classification probabilities

The probability of GHS class 1, following 20 random partitions of the SARA-ICE database, is plotted for each OECD benchmark chemical in Figure 1. 80 chemicals were assigned GHS class 1 in every partition, 20 chemicals were assigned NC in every partition and 57 chemicals were consistently assigned inconclusive classifications. The percentage of chemicals whose classification was stable under the random partitions is 80%. The remaining 39 chemicals had a classification that changed in at least one partition (20%). Classification changes typically occur when the probability of GHS class 1 is close to the decision boundaries of P(1)>0.8 (call GHS class 1) or P(NC)>0.8 (call NC) (equivalent to requiring P(1)<0.2). If a change occurs, the change is between an inconclusive and conclusive call. There are no examples of a conclusive call of 1 changing to a conclusive call of NC or vice versa.


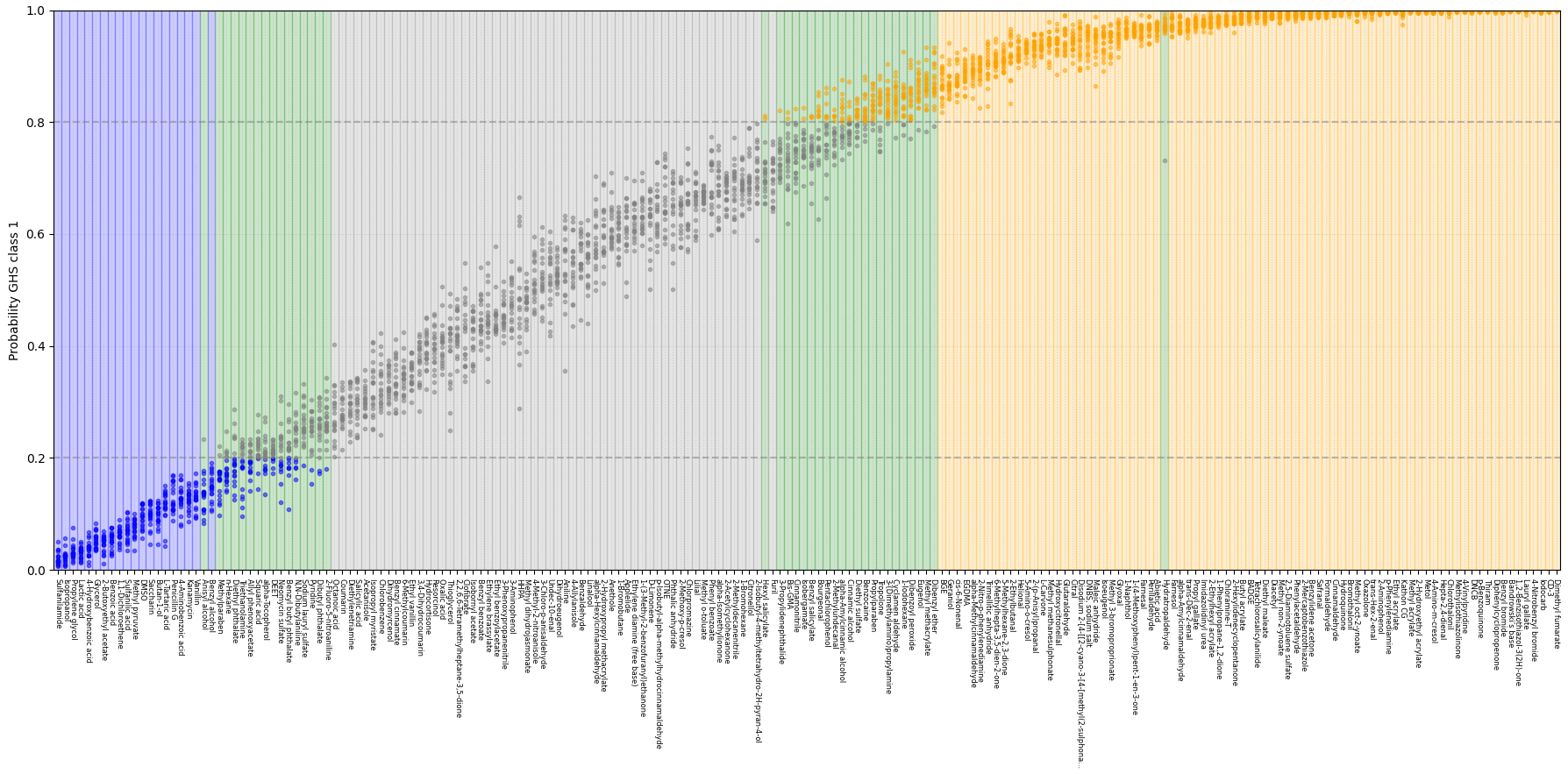


Figure 1 Probability of GHS class 1 obtained under each repeat of the cross-validation procedure. Blue bars indicate chemicals whose binary classification is NC following every random partition of the OECD benchmark dataset. Orange bars indicate chemicals whose binary classification is consistently 1. Grey indicates consistent inconclusive calls and green indicate chemicals whose classification changes in each at least one partition.

### Variability in summary classification statistics

Overall binary classification metrics for conclusive calls, summarised in terms of sensitivity, specificity, and balanced accuracy, were calculated for each random partition of the OECD benchmark chemical set. These metrics are plotted alongside the performance metric obtained when training on the full dataset, in Figure 2. The standard deviation of the binary sensitivity is less than 1 percentage point for both human and LLNA benchmarks, indicated that this metric is reasonably stable with respect to randomly throwing out 10% of the benchmark chemicals during training. Specificity for the LLNA benchmarks has a standard deviation less than 2 percentage points. The variation is higher for the human benchmarks, but this variation is driven by the relatively small number of chemicals with a human classification of NC.


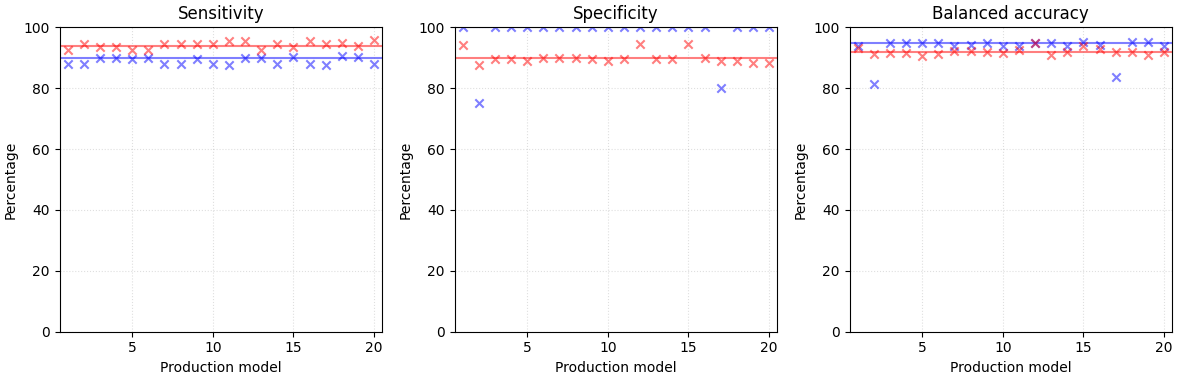


Figure 2 Sensitivity, specificity, and balanced accuracy for binary GHS classifications for human (blue) and LLNA (red) benchmarks calculated following each random 10-fold partition of the OECD benchmark chemical set. The horizontal line indicates the computed statistics when the production model is trained on all available data in the SARA-ICE database, i.e., the statistics presented in Table 1.

Classification metrics for subcategory predictions calculated within each iteration are plotted in Figure 3. Variability of the statistics across random partitions is marginally higher for most categories but more noticeably for subcategory 1A and the specificity of subcategory 1B. There is a slight bias with respect to the statistics presented in Table 2: the mean difference between the performance metrics computed without removing data to the mean performance across each cross-validation run is approximately 2 percentage points (1.5 for LLNA and 2.5 for human). Average balanced accuracy for subcategory classification, averaged across cross validation repeats is approximately 80.5% for both human and LLNA benchmarks. This is slightly lower than percentages of 83% and 82% reported in Table 2.


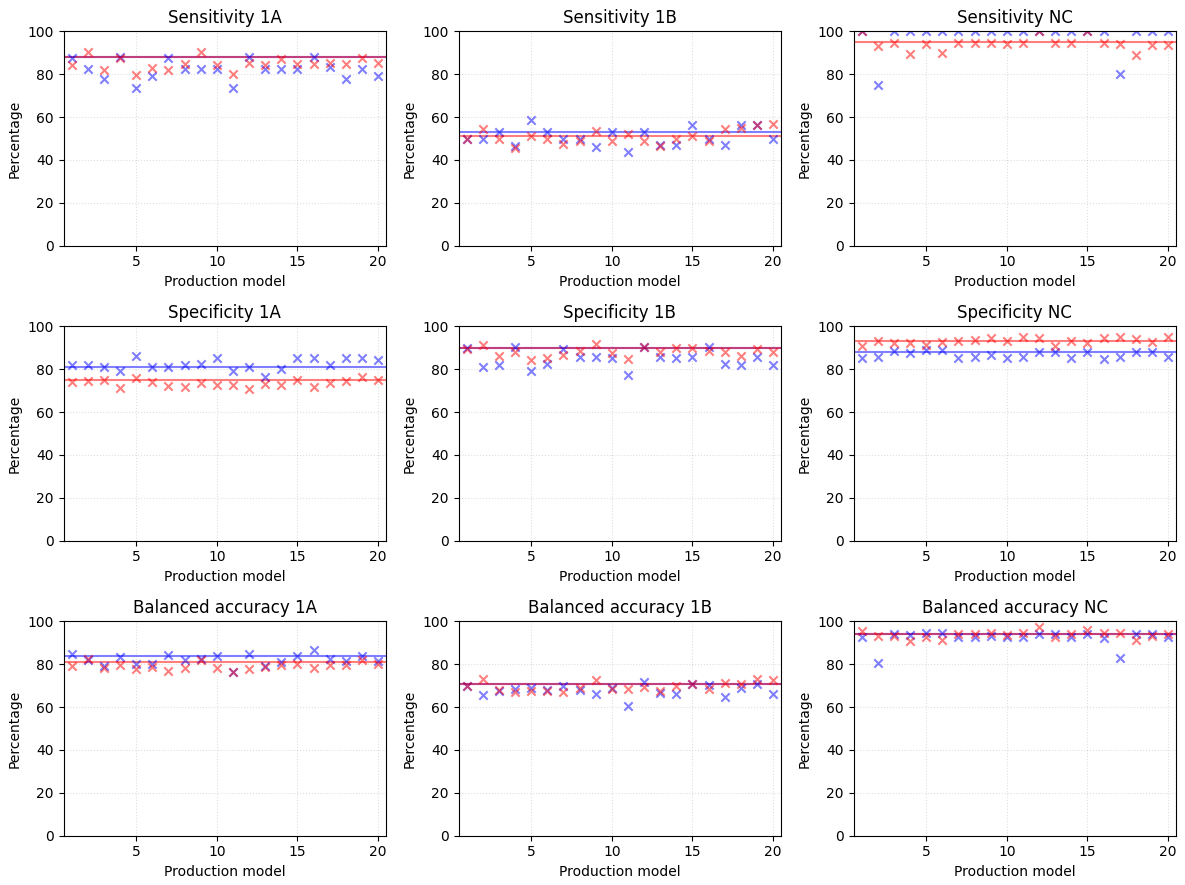


Figure 3 Sensitivity, specificity, and balanced accuracy for subcategory GHS classifications for human (blue) and LLNA (red) benchmarks calculated following each random 10-fold partition of the OECD benchmark chemical set. The horizontal line indicates the computed statistics are computed from the production model trained using the full SARA-ICE database, i.e., the statistics presented in Table 2.

### Estimation of distribution of performance statistics

The cross-validation procedure performed above is computationally expensive and therefore we are limited in the number of times it can be repeated. To estimate the distribution of classification performance statistics, we aim to characterise the distribution of GHS classification probabilities obtained following the 20 repeats of the cross-validation procedure using a generative statistical model. This model is then used to sample 1,000 samples of the GHS classification probabilities and for each set, we calculate classification performance statistics against the OECD benchmark dataset.

#### Statistical model for distribution of GHS probabilities

The following statistical model is applied independently to each OECD benchmark chemical. SARA-ICE classification probabilities are estimated using Monte Carlo approach. The actual output of the model is a count $k_{C}$ (out of $N=1,000$) for classes $C=\{\text{1A},\text{1B},\text{NC}\}$. The reported probability for each class is simply $p_{C}=k_{C}/N$.

Let be the $i$th count for GHS class $C$ obtained from the cross-validation procedure with $i\in\{1,\ldots,20\}$. The vector of counts is modelled as drawn from a multinomial distribution

$$k_{i}\sim\text{MultiNomial}\left(p_{i}\right)$$

where the probability simplex is the latent (unobservable) true SARA-ICE classification probability. This probability is treated as a random variable within this statistical model. The probability vector can be completed specified in terms of unknown probabilities and by defining

We model the distribution of and across repeats of the cross-validation procedure by assuming the logit transform of these probabilities following skew-normal distributions:

The distribution parameters assigned the weakly informative prior distributions

$$\begin{matrix} \xi_{1},\xi_{\text{1A}\mid1}\sim\text{Normal}\left(0,5\right) , \\ \omega_{1},\omega_{\text{1A}\mid1}\sim\text{HalfNormal}\left(0,1\right), \\ \alpha_{1},\alpha_{\text{1A}\mid1}\sim\text{Normal}\left(0,5\right) . \end{matrix}$$

### Results

The statistical model in the previous section was fit to the set of 20 SARA-ICE classification probabilities for each OECD benchmark chemical. The posterior distribution of the parameters describing the distribution of classification probabilities was used to sample 1,000 sets of SARA-ICE classification probabilities for each chemical. In Figure 4 we compare the distribution of the simulated classification probabilities against those obtained from the 20 runs of the cross-validation procedure for 20 randomly selected chemicals. This figure serves as a posterior predictive check for the model and indicates that the model is flexible enough to describe the variance and skew in the classification probabilities for each chemical. Plots for all chemicals are provided in the supplementary PDF “Posterior predictive GHS probabilities”.

The model was used to sample 1,000 sets of GHS classification probabilities for each chemical, which in turn allowed us to calculate 1,000 sets of classification performance metrics against the OECD benchmark chemical dataset. These are plotted in Figure 5, completely analogous to those plotted above in Figure 2 and Figure 3. Since the number of benchmarks is finite, the number of possible classification performance metrics is finite. This is most noticeable for the estimates of human binary specificity, which, due to the small number chemicals classified as human NC, only takes values in the set 67%, 75%, 80%, 83.3% and 100% with the frequencies 3/1000, 15/1000, 64/1000, 2/1000 and 916/1000. A specificity as low as 67% is therefore possible, but implausible (we would expect to have to repeat the cross-validation procedure over 300 times to observe it this low). Conversely, we expect it to be 100% in the majority (92%) of repeats of the cross-validation procedure.

Histograms of balanced accuracy for the binary classification and average balanced accuracy for the subcategory classification are presented in Figure 6. Balanced accuracy for binary classifications against LLNA benchmarks exceeds 90% in most simulations, with a mean value of 92%. Mean balanced accuracy against human benchmarks is 94%, but the distribution is a bimodal with a lower mode at around 85% which arises due to the infrequent drop in specificity discussed above. The mean of average balanced accuracy samples for the subcategory classification is approximately 81% for both species. Mean estimates are similar to those computed using the full SARA-ICE database in Table 1 and Table 2.


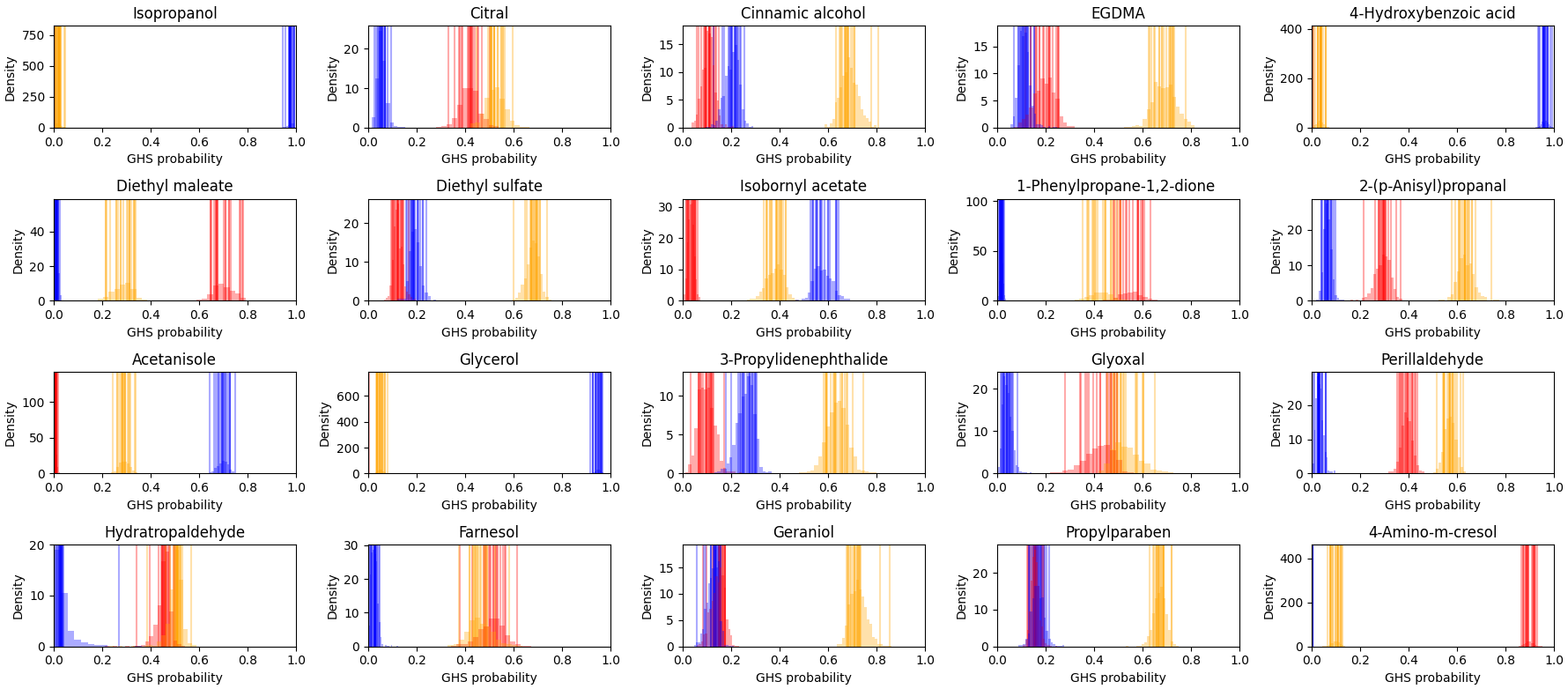


Figure 4 Vertical lines indicate GHS classification probabilities for 20 randomly selected chemicals computed from each of the 20 repeats of the 10-fold cross validation procedure. Histograms are estimated from 1,000 simulated probabilities estimated from the statistical model used to describe the variability of these probabilities. Red: GHS 1A, orange: GHS 1B, blue: NC. Plots for all chemicals are provided in the supplementary PDF “Posterior predictive GHS probabilities”.


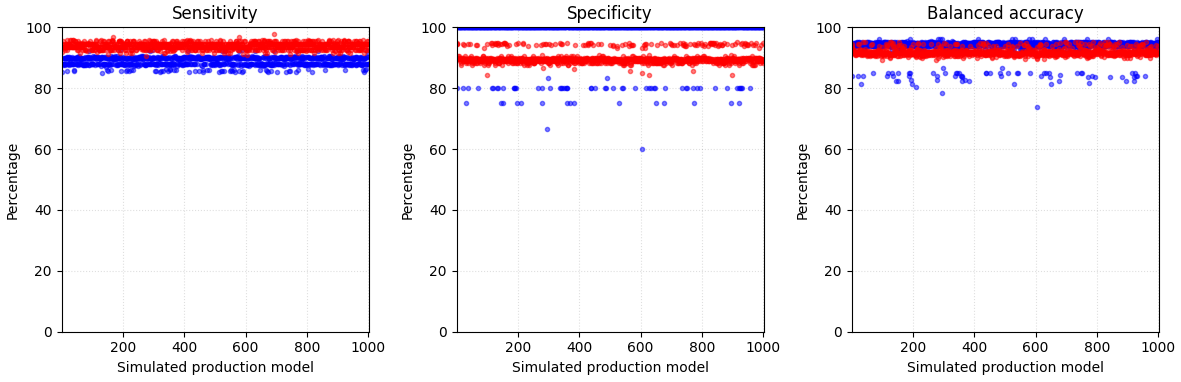


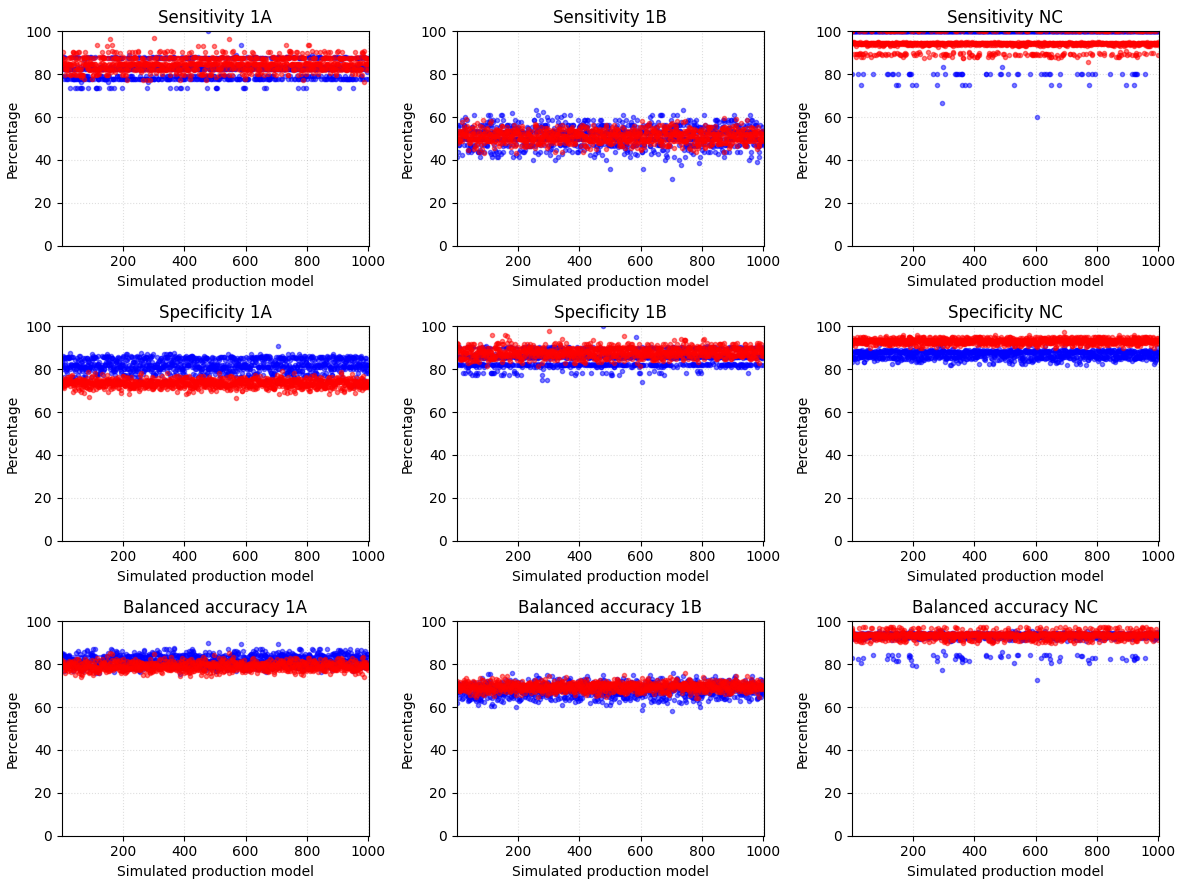


Figure 5 Simulated classification performance against the OECD benchmark dataset obtained from statistical extrapolation of the 20 actual estimates obtained in the previous section. Blue: human benchmarks, red: LLNA benchmarks.


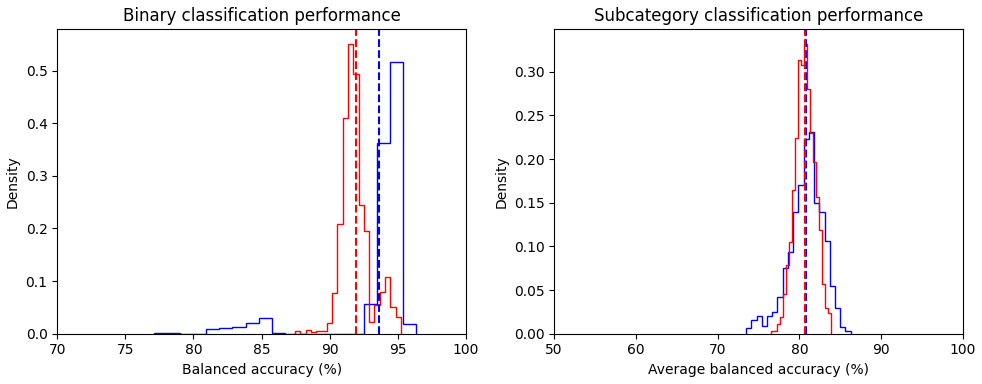


Figure 6 Simulated distribution of overall classification performance (balanced accuracy for binary classification, averaged balanced accuracy for subcategory classification performance) against human (blue) and LLNA (red) benchmarks.

## Supplementary Documents

GHS classifications and underlying probabilities obtained using all available data in the SARA-ICE database are provided in the supplementary file “GHS_analysis_full_database.xlsx” on the “all_available_data” sheet. The last columns indicate contain counts of each SARA-ICE input underpinning the obtained classification for each chemical. The second sheet, “oecd_nam_inputs_sara_prod”, contains GHS classifications and probabilities obtained by using OECD benchmark NAM data to generate classification with SARA-ICE production with parameters estimated from the full database.

The supplementary file “GHS_analysis_cross_validation.xlsx” contains GHS classifications and probabilities obtained from each of the 20 repeats of the cross-validation procedure.

## References

Geier, J., Ballmer‐Weber, B., Buhl, T., Rieker‐Schwienbacher, J., Mahler, V., Dickel, H., . . . Grabbe, J. (2022). Is benzyl alcohol a significant contact sensitizer? *Journal of the European Academy of Dermatology and Venereology, 36*(6), 866-872.

Natsch, A., & Gerberick, G. F. (2022). Integrated skin sensitization assessment based on OECD methods (I): Deriving a point of departure for risk assessment. *ALTEX-Alternatives to animal experimentation, 39*(4), 636-646.

OECD. (2021). *SUPPORTING DOCUMENT TO THE OECD GUIDELINE 497 ON DEFINED APPROACHES*

*FOR SKIN SENSITISATION*.
