# Supplementary material for: The skin allergy risk assessment-integrated chemical environment (SARA-ICE) defined approach to derive points of departure for skin sensitization
Source: Curr Res Toxicol. 2024 Dec 14;8:100205. doi: 10.1016/j.crtox.2024.100205 (PMC11719337; doi:10.1016/j.crtox.2024.100205)
Supplement: Supplementary Data 3 [file mmc3.docx]

Supplemental Information Tables

Supplemental Table 1 POD derived from ANN models for isothiazolinones

| **Chemical**  **Name** | ANN 1 | | ANN 2 | |
| --- | --- | --- | --- | --- |
|  | **Predicted EC3 (%)^3^** | **(µg/cm^2^)** | **Predicted EC3 (%)^3^** | **(µg/cm^2^)** |
| DCOIT | 0.0566 | 14 | 0.023 | 5.75 |
| CMIT/MIT | 0.121 | 30.25 | 0.492 | 123 |
| OIT | 0.0569 | 14.3 | 0.015 | 3.75 |
| MIT | 1.775 | 443.75 | 0.826 | 206.5 |
| BIT | 0.934 | 233.5 | 0.341 | 85.25 |
| BBIT | 0.148 | 37 | 0.061 | 15.25 |

ANN values derived in *Strickland et al., 2022* and coverted to dose per skin area using a factor of 250 as described in *Greim et al., 2003.*

Supplemental Table 2 Input data for the isothiazolinones.

| **Study Type** | **Chemical** | | | | | | **Source** |
| --- | --- | --- | --- | --- | --- | --- | --- |
|  | **CMIT/MIT** | **MIT** | **DCOIT** | **OIT** | **BIT** | **BBIT** |  |
| **DPRA** | Cysteine depletion: 100%  Lysine depletion: 10.6% | Cysteine depletion: 100%  Lysine depletion: 0% | Cysteine depletion: 100%  Lysine depletion: 11.6% | Cysteine depletion: 100%  Lysine depletion: 1.3% | Cysteine depletion: 100%  Lysine depletion: 0% | Cysteine depletion: 100%  Lysine depletion: 0% | NICEATM IT report, Appendix A, Table 2 |
| **KeratinoSens** | EC_1.5_: 3.41 µM  IC_50_: 19.9 µM | EC_1.5_: 9.54 µM  IC_50_: 108 µM | EC_1.5_: 1.32 µM  IC_50_: 4.65 µM | EC_1.5_: 2.19 µM  IC_50_: 12.7 µM | EC_1.5_: 3.14 µM  IC_50_: 57.8 µM | EC_1.5_: 3.84 µM  IC_50_: 53.0 µM | NICEATM IT report, Appendix A, Table 5 |
| **h-Clat** | CD54 EC_200_: 2.63 µg ml^-1^  CD86 EC_150_: 2.81 µg ml^-1^  CV_75_: 3.04 µg ml^-1^ | CD54 EC_200_: 11.6 µg ml^-1^  CD86 EC_150_: 11.8 µg ml^-1^  CV_75_: 24.6 µg ml^-1^ | CD54 EC_200_: 0.92 µg ml^-1^  CD86 EC_150_: >1.08^1^ µg ml^-1^  CV_75_: 0.9 µg ml^-1^ | CD54 EC_200_: 0.95 µg ml^-1^  CD86 EC_150_: 7.26 µg ml^-1^  CV_75_: 8.8 µg ml^-1^ | CD54 EC_200_: 7.63 µg ml^-1^  CD86 EC_150_: 7.84 µg ml^-1^  CV_75_: 13.1 µg ml^-1^ | CD54 EC_200_: 3.01 µg ml^-1^  CD86 EC_150_: 3.15 µg ml^-1^  CV_75_: 3.3 µg ml^-1^ | NICEATM IT report, Appendix A, Tables 7 & 8 |
| **LLNA** | EC3 = 0.0065%,  EC3 = 0.0082%,  EC3 = 0.063%,  EC3 = 0.0076%,  EC3 = 0.0049%,  EC3 = 0.0075%,  EC3 = 0.0075%,  EC3 = 0.0068%,  EC3 = 0.048%, | EC3 = 0.4%,  EC3 = 0.86%,  EC3 = 2.2%,  EC3 > 4.5% | EC3 = 0.011%,  EC3 = 0.0041% | EC3 = 0.66%,  EC3 = 0.2%,  EC3 = 0.2%,  EC3 = 0.33% | EC3 = 32.4%,  EC3 = 4.8%,  EC3 = 2.3%,  EC3 = 29%,  EC3 = 1.8%,  EC3 = 2.2%,  EC3 = 1.5% |  | NICEATM IT report, Appendix C |
| **HPPT** | (DSA = 2.9 µg cm^-2^, N_tested_ = 45, N_sensitised_ = 2),  (DSA = 39 µg cm^-2^, N_tested_ = 96, N_sensitised_ = 0),  (DSA = 79 µg cm^-2^, N_tested_ = 104, N_sensitised_ = 2),  (DSA = 0.42 µg cm^-2^, N_tested_ = 416, N_sensitised_ = 0),  (DSA = 0.5 µg cm^-2^, N_tested_ = 103, N_sensitised_ = 0),  (DSA = 0.75 µg cm^-2^, N_tested_ = 184, N_sensitised_ = 0),  (DSA = 0.83 µg cm^-2^, N_tested_ = 602, N_sensitised_ = 0),  (DSA = 1.04 µg cm^-2^, N_tested_ = 84, N_sensitised_ = 1),  (DSA = 1.25 µg cm^-2^, N_tested_ = 200, N_sensitised_ = 0),  (DSA = 1.34 µg cm^-2^, N_tested_ = 189, N_sensitised_ = 2),  (DSA = 2.5 µg cm^-2^, N_tested_ = 109, N_sensitised_ = 0),  (DSA = 5 µg cm^-2^, N_tested_ = 116, N_sensitised_ = 5),  (DSA = 7.5 µg cm^-2^, N_tested_ = 196, N_sensitised_ = 7) | (DSA = 10 µg cm^-2^, N_tested_ = 100, N_sensitised_ = 0),  (DSA = 15 µg cm^-2^, N_tested_ = 98, N_sensitised_ = 0),  (DSA = 20 µg cm^-2^, N_tested_ = 116, N_sensitised_ = 1),  (DSA = 25 µg cm^-2^, N_tested_ = 210, N_sensitised_ = 1),  (DSA = 30 µg cm^-2^, N_tested_ = 214, N_sensitised_ = 0),  (DSA = 30 µg cm^-2^, N_tested_ = 75, N_sensitised_ = 0) |  |  | (DSA = 45 µg cm^-2^, N_tested_ = 54, N_sensitised_ = 0),  (DSA = 91 µg cm^-2^, N_tested_ = 58, N_sensitised_ = 5) |  | Strickland et al., 2023 |

NICEATM IT Report (https://ntp.niehs.nih.gov/sites/default/files/iccvam/methods/immunotox/it/niceatm-it-report-june2020-final-wappx-508.pdf)

Strickland, J. *et al.* A database of human predictive patch test data for skin sensitization. *Arch Toxicol* (2023) doi:10.1007/s00204-023-03530-3.
